# Recent Progress in Nucleic Acid Pulmonary Delivery toward Overcoming Physiological Barriers and Improving Transfection Efficiency

**DOI:** 10.1002/advs.202309748

**Published:** 2024-03-09

**Authors:** Qiyue Wang, Chaozhi Bu, Qihao Dai, Jinhua Chen, Ruitao Zhang, Xiaomin Zheng, Hao Ren, Xiaofei Xin, Xueming Li

**Affiliations:** ^1^ School of Pharmaceutical Science Nanjing Tech University Nanjing 211816 China; ^2^ NMPA Key Laboratory for Research and Evaluation of Pharmaceutical Preparation and Excipients Nanjing 210009 China; ^3^ Wuxi Maternity and Child Health Care Hospital Affiliated Women's Hospital of Jiangnan University Wuxi 214002 China; ^4^ Center for Research Development and Evaluation of Pharmaceutical Excipients and Generic Drugs, Department of Pharmaceutics China Pharmaceutical University Nanjing 210009 China

**Keywords:** delivery vectors, inhaled materials, nucleic acids, pulmonary bioavailability, pulmonary delivery

## Abstract

Pulmonary delivery of therapeutic agents has been considered the desirable administration route for local lung disease treatment. As the latest generation of therapeutic agents, nucleic acid has been gradually developed as gene therapy for local diseases such as asthma, chronic obstructive pulmonary diseases, and lung fibrosis. The features of nucleic acid, specific physiological structure, and pathophysiological barriers of the respiratory tract have strongly affected the delivery efficiency and pulmonary bioavailability of nucleic acid, directly related to the treatment outcomes. The development of pharmaceutics and material science provides the potential for highly effective pulmonary medicine delivery. In this review, the key factors and barriers are first introduced that affect the pulmonary delivery and bioavailability of nucleic acids. The advanced inhaled materials for nucleic acid delivery are further summarized. The recent progress of platform designs for improving the pulmonary delivery efficiency of nucleic acids and their therapeutic outcomes have been systematically analyzed, with the application and the perspectives of advanced vectors for pulmonary gene delivery.

## Graphical abstract

Pulmonary gene delivery has obvious advantages for the treatment of inherited lung diseases, while still being obstracted by lacking of the effective delivery platforms. The development of pulmonary physiology, inhalable carrier materials, and their transport strategies for therapeutic nucleic acids helps improving the transfection efficiency of gene medicine.

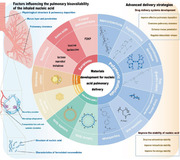

## 1 Introduction

As a critical organ of the respiratory system, the lung has its unique physiological structure, which has been investigated as the particular drug delivery route for local or systemic drug delivery. The large air‐blood gas exchange area, thin epithelial membrane, and avoidance of first‐pass effects made the pulmonary drug administration an attractive systemic drug delivery route.^[^ ^1^ ^]^ For the pulmonary local disease treatment, improving the drug accumulation in lung tissue or even in targeted pulmonary subtype cells could be more effective. Several drug delivery systems have been designed with an acceptable transfection efficiency in pulmonary capillary endothelium targeting via systemic administration.^[^ ^2^, ^3^ ^]^ Moreover, pulmonary cell‐specific drug targeting has also been reported via verifying the structure of delivery vehicles or their surface modification.^[^ ^4^, ^5^ ^]^ However, the unique physiological barrier of the lungs suggested the importance of cell‐specific drug target delivery route selection. The drug administration orally or by injection is mainly designed for endothelial cells targeting delivery but hardly achieves enough pulmonary accumulation, especially in bronchial epithelial cells, alveolar epithelial cells, and resident alveolar macrophages. Therefore, the pulmonary delivery route could be the first option for non‐endothelial cell targeting due to direct contact with the drug carriers. Moreover, drug inhalation provides high lung‐targeted drug accumulation and pulmonary bioavailability, which is defined as a dose deposited in the lung and uptake by respiratory epithelial cells, more suitable for local pulmonary disease treatment. Pulmonary drug delivery platforms have been developed for the effective therapeutic of asthma, chronic obstructive pulmonary disease (COPD), pulmonary fibrosis, lung bacterial infection, or systemic administration of protein drugs.^[^ ^6^, ^7^, ^8^, ^9^, ^10^, ^11^ ^]^ With the outbreak of COVID‐19, the messenger RNA (mRNA) vaccine has been successfully commercialized, drawing a lot of attention to the development of nucleic acid drug delivery systems from pharmaceutical companies and scientific researchers.^[^ ^12^, ^13^, ^14^ ^]^ Therefore, pulmonary delivery of nucleic acid has been considered the promising route for vaccine development and local disease gene therapy.

Gene therapy could directly affect the cell cycle or metabolism, offering the chance for a complete cure of diseases. Nucleic acids such as plasmids DNA (pDNA), mRNA, small interfering RNA (siRNA), microRNA (miRNA), and circular RNA (circRNA) could directly or indirectly regulate the key signaling pathways in specific diseases via producing gene expression, gene silencing, and gene deletion followed by disrupt their coded proteins expression.^[^ ^15^, ^16^, ^17^ ^]^ Even though therapeutic nucleic acid has exhibited potential in tumor precision therapy, genetic disease therapy, and vaccination, their effective delivery to the target cells as the major obstacle strictly restricts their development and application in the clinic.^[^ ^18^, ^19^ ^]^ Naked nucleic acid was unstable due to its long chains constructed with a series of nucleotide units that easily degraded. Therefore, several technologies, such as RNA modification, have been developed to improve the stability of gene drugs.^[^ ^20^ ^]^ Moreover, designing gene carriers to load therapeutic nucleic acids to enhance their stability and delivery efficiency is also being considered to overcome these problems. With material science and nanotechnology development, nano‐carrier types have been designed for nucleic acid targeted delivery with improved transfection efficiency.

The pulmonary local disease treatment and targeted nucleic acid delivery system have been focused on as the next attractive direction for pharmaceutical research.^[^ ^21^ ^]^ The inhaler device was always needed for the nucleic acid pulmonary deposition to the target areas. The inhalation devices were divided into several types: nebulizers, metered dose inhalers (MDIs), and dry powder inhalers (DPIs).^[^ ^22^ ^]^ The nebulizers and MDIs were mainly used for formulations formed in solution or suspension, while the DPIs were designed for the formulations in powder form. The nucleic acid could be formulated as solution or powder form with the carrier's encapsulation. Water could be the important factor inducing their degradation, which suggests the dry powder form should be the optimal formulation for storage.^[^ ^1^ ^]^ Moreover, the suitable formulation design and devices were another main factor that affected the fate of nucleic acid inhalation. The deposited drugs could be cleared by the special protection system of the lungs and reduce their pulmonary bioavailability. To improve the stability of nucleic acid nanomedicine and achieve their effective delivery, developing multifunctional inhalable carriers and formulations was the major investigation pharmaceutics expected to accomplish.

In this review, we start by presenting the specific pulmonary structure and delivery barriers that affect nucleic acid nanomedicine pulmonary delivery. The key characteristics that affect the formulation deposition efficiency are further introduced. We further systematically summarize the inhalable biosafety materials being investigated for nucleic acid pulmonary delivery preclinic to illustrate the design direction of the gene delivery system. Finally, the nucleic acid formulation design strategies for overcoming the separate delivery barrier after pulmonary administration are summarized, and their potential application in clinical trials is summarized.

## 2 Factors Influencing the Pulmonary Bioavailability of the Inhaled Nucleic Acid

**Figure 1:**
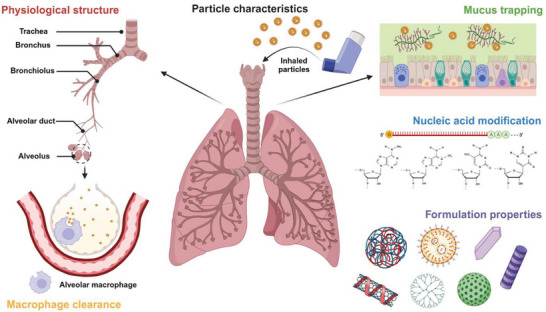
Factors influencing the pulmonary bioavailability of the inhaled nucleic acid. Created with BioRender.com.

Formulated nucleic acid must overcome several biological barriers before successful cell transfection after inhaling.^[^ ^23^ ^]^ Physiological structure and barriers of the respiratory system could influence the pulmonary bioavailability of the inhaled nucleic acid by intervening in the deposition, clearance, mucus penetration, cellular uptake, and lysosomal escape process. Moreover, the formulation characters and specific features of nucleic acid could also affect the transfection efficiency by regulating the stability of nucleic acid. Here, we briefly introduce several of those factors and illustrate how they affect the pulmonary bioavailability of nucleic acid (**Figure** **1**).

### 2.1 Physiological Structure and Pulmonary Deposition

The lungs are the critical organ of the respiratory system and carry out the function of gas exchange. The lung is composed of a series of bifurcations of the trachea and alveolus, exhibited as an inverted tree.^[^ ^24^ ^]^ Starting from the trachea, the respiratory tract was gradually bifurcated by about 23 bifurcations and ended as a dilated alveolus. The first 15 grades of airway branches function as airflow conduction, which exhibits a similar structure composed of the epithelium (Goblet cells and Kulchitsky cells), lamina propria (Pulmonary mucosa‐associated lymphoid tissue and epithelium support), and respiratory smooth muscle layer. Starting from the 16th bifurcation, the bronchi were progressively replaced with a thin layer of alveolar epithelial cells with the function of gas exchange.^[^ ^25^ ^]^

With the respiratory airway bifurcation, the airway diameter progressively reduces, and the airway resistance gradually increases, which, combined with the bifurcation amount increment, becomes the main reason for the large inhaled particle's local deposition.^[^ ^26^ ^]^ With an aerodynamic diameter larger than 5 µm, the inhaled particles hardly move forward with the airflow due to inertia and easily collide with the respiratory tract and are deposited. The specific physiological structure not only prevents the absorption of harmful pollutants by the lung but also forms a barrier for the inhaled therapeutic agents to be deposited deep into the lung. Therefore, most of the large particles could be blocked by the throat and bifurcations, which significantly reduce the pulmonary bioavailability of the inhaled nucleic acid.^[^ ^27^ ^]^

The efficiency of drug absorption is strongly related to their deposition area. The epithelial cells in airflow conduction areas form tight junctions and barrier the penetration of medicines deposited, significantly affecting the systemically acting drugs. It's been reported the transportation efficiency of biological drugs is related to their molecular weight, and the hydrophilic proteins smaller than 40 kDa could pass the barrier via the paracellular pathway.^[^ ^28^ ^]^ For the drugs deposited in the alveoli, the thin alveolar epithelium layer and pulmonary surfactant help their absorption via cellular and paracellular pathways.

The changes in the physiological structure of the respiratory tract caused by pulmonary diseases can also affect the pulmonary bioavailability of inhaled nanomedicine. Idiopathic pulmonary fibrosis and COPD patients with low respiratory function only achieve gas exchange under a low breathing air velocity, which reduces deep particle deposition and, therefore, low pulmonary bioavailability.^[^ ^29^ ^]^ Cystic fibrosis, a genetic disease with cystic fibrosis transmembrane conductance regulator mutation, could induce the thickened and sticky mucus layer, which blocks the nucleic acid‐loaded particle penetration and inhibits the mucociliary escalator.^[^ ^30^ ^]^ Non‐cystic fibrosis and pulmonary infection could also affect the therapeutic gene's successful delivery via induced airway inflammation and improved pulmonary clearance by macrophage phagocytosis and mucus restriction.^[^ ^31^ ^]^

### 2.2 Mucus Layer and Penetration

**Figure 2:**
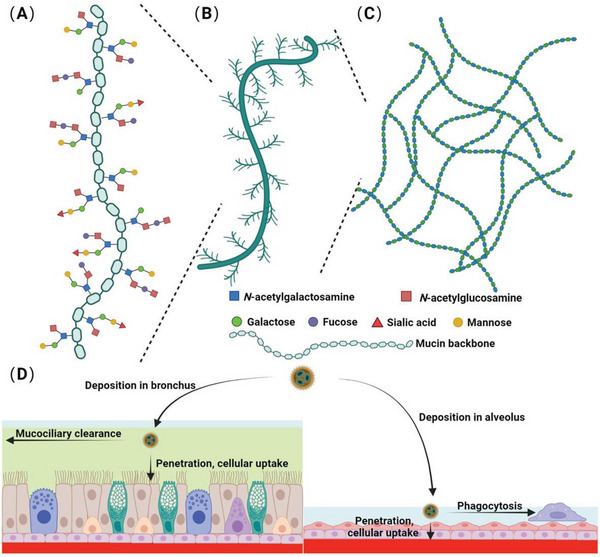
The macromolecular structure of airway mucins and the fate of pulmonary deposited particles. A) The mucin backbone has various glycosylation modifications. B) The mucin monomer contains serine‐, threonine‐, and proline‐rich amino acids in the backbone. C) The mucin monomer assembles into multimer states that exist as linear, branched, or side‐linked structures via disulfide bonds. D) The fate of aerosol drugs after deposition in the bronchus or alveolus via mucociliary clearance, macrophage phagocytosis, or uptake into epithelial cells or systemic circulation. Created with BioRender.com.

The surface of the respiratory tract wall contains different types of secretory cells that could produce various mucus compositions. The respiratory mucus was composed of water (90–98%), polymerized mucins (2–5%), and other biological molecules such as defensins, cellular lysate, and immunoglobulin A, which all being synthesized and secreted into the airways, followed by forming a hydrogel like mucus layer covering the surface of the respiratory tract.^[^ ^32^ ^]^ The mucin proteoglycans were a proline‐, serine‐, and threonine‐rich amino acid line chain grafted with O‐glycan structures side chain formed by N‐acetylgalactosamine, galactose, fucose, sialic acid, mannose, and N‐ acetylglucosamine.^[^ ^33^ ^]^ The C‐terminus and N‐terminus with rich sulfhydryl bonds of monomer mucins could assemble and form a dynamic network structure via breaking or forming the disulfide bonds, electrostatic interaction, and hydrophobic force (**Figure** **2**). Based on the component of the pulmonary mucus, the naked nucleic acid was unsuitable for inhalation as it faced the nuclease degradation from the cellular lysate.^[^ ^34^ ^]^

Due to the assembled supramolecular structure, the mucus exhibited a hydrogel with viscosity thousands of times higher than water. The long mucin proteoglycans chain intertwined and formed hydrophobic domains and hydrophilic channels, which might be the reasons for the penetrating inhibition of inhaled molecules.^[^ ^35^ ^]^ The hydrophobic molecules always hindered penetration in mucus compared to water, which was caused by the interactions between their hydrophobic groups and mucus hydrophobic domains. Usually, hydrophilic macromolecules such as naked nucleic acid proteins delay their mucus penetration because of their high viscosity, which reduces the penetrating rate and causes the drug to be cleared with the mucus. However, several research groups have confirmed that proteins or antibodies could rapidly diffuse through mucus via the matrix low viscosity channels when the particle agents were smaller than the channel diameter. The structure of pulmonary mucus has been reported by Meziu et al., who found no visualizable porous structure was observed in pulmonary mucus, suggesting the pulmonary mucus contains low viscosity channels for particles smaller than 200 nm long‐distance diffusion.^[^ ^36^ ^]^ The research from other groups also confirms these features, drawing attention to particle size and structure optimization in inhalation development.

Moreover, the pulmonary mucus also exhibited as the sticky net that trapped the compounds, virus, and inhaled nanoparticles, followed by mucociliary clearance. The inhaled particles could interact with polymerized mucins by forming intermolecular forces. The free sulfhydryl of the inhaled compounds could be oxidized and form disulfide bonds with mucins, which lock them in and reduce their penetration.^[^ ^37^ ^]^ In addition, the carboxyl groups on the mucin proteoglycans exhibited negative charges, which readily combine with the vehicles with positive charges, especially nanocarriers for gene delivery, and trap them via electrostatic interaction. Several researchers have confirmed the particles with negative or neutral surface charges exhibited a rapid mucus penetrating rate compared to those with positive surface charges.^[^ ^38^, ^39^ ^]^ The hydrophobic domains of mucin proteoglycans also show a high affinity called a hydrophobic force to the hydrophobic regions of the foreign particles, further inhibiting their mucus penetration. Several research groups have confirmed that mucus could strongly interact with the hydrophobic area of particles and trap them inside, even which has modified surface and carrier negative charges.^[^ ^40^, ^41^ ^]^ The polymeric nanoparticles design for nucleic acid pulmonary delivery could be strongly affected by both electrostatic interaction and hydrophobic force as the delivery materials always contain a hydrophobic block (polyester or polyanhydrides) and cationic surface (polyethyleneimine, chitosan, or polypeptide) for particle self‐assemble and gene loading, respectively. Therefore, lots of effort is still being paid for the mucus low adhesive nanomaterials design and drug delivery system development.

### 2.3 Pulmonary Clearance

Without a breathing chance, the deposited nanomedicines immediately need to face the clearance effect of other respiratory physiological barriers, such as mucociliary escalator and macrophage phagocytosis. Moreover, some inhaled nanomedicines rapidly translocate into cells, blood, or lymph, which may reduce their pulmonary retention and treatment efficiency for local therapy. The clearance pathway can also be an important factor affecting the pulmonary bioavailability of inhaled nanomedicine (Figure 2).

#### 2.3.1 Mucociliary Escalator

The airway epithelium in the upper and middle respiratory exists a cilia structure for effectively transporting secreted mucus out of the lung.^[^ ^42^ ^]^ The regulation of ciliary beating continuously pushes mucus from the bronchioles to the trachea and then to the esophagus, also being named mucociliary escalator, a self‐clearing process of pulmonary. The cilia work with mucus and effectively removes foreign objects from the lungs. The mucin proteoglycans could capture many of the deposited nanomedicine particles and will be cleared with the mucus as a significant factor in reducing their pulmonary bioavailability and therapeutic efficiency.^[^ ^43^ ^]^ The existence of the mucociliary escalator prompted scientists to design an intelligent pulmonary drug delivery system that could avoid mucus bondage.

#### 2.3.2 Macrophage Phagocytosis

With the airway narrow in the deep lung, the mucus layer gets thinner, and the mucociliary escalator does not exist in the bronchioles and alveolus. However, that doesn't mean inhaled nanomedicine could be directly absorbed into local cells or cycle systems. Macrophage phagocytosis is another important mechanism for the pulmonary clearance of deposited particles and is considered the primary clearance mechanism in the alveoli.^[^ ^31^, ^44^ ^]^ The alveolar macrophages originate from the circulating fetal monocytes when the newborn first breath, which is then differentiated and induced by GM‐CSF secretion by alveolar type II cells.^[^ ^45^ ^]^ The alveolar macrophage acts as an essential immune cell in the lung to eliminate the inhaled pollutants, pathogens, and foreign particles deposited in the alveoli. The particle size of deposited particles has been considered a key characteristic of macrophage‐selective phagocytosis.^[^ ^46^ ^]^ Several researchers have demonstrated that particles with a geometric size of ≈1–3 µm were the preferred targets for phagocytosis of alveolar macrophages.^[^ ^47^ ^]^ The pulmonary surfactant secreted by alveolar type II cells covered on the surface of epithelial cells acts not only to reduce surface tension but also to participate in macrophage‐based alveolar clearance. The deposited particles or macromolecules would be coated by the pulmonary surfactant with surfactant proteins SP‐A or SP‐D, then recognized by alveolar macrophages and induce phagocytosis, which reduced pulmonary bioavailability and might cause an unnecessary immune response.^[^ ^48^, ^49^ ^]^

#### 2.3.3 Transportation into Cells and Systemic Circulation

Transportation of nanomedicines into the epithelial cells or cycle system is the two crucial pathways for inhaled therapeutic agents' efficacy and would be processed simultaneously. With the different therapeutic targets, investigators made lots of efforts to either increase the agent's uptake by epithelial cells for local enrichment or improve the agents' transport through epithelial cells to circulation. For circulation delivery, inhaled agents need to move rapidly across epithelial cells, which act as a strong absorption barrier. Improving the dissolution of inhaled nanomedicines could help their absorption via diffusion and reduce macrophage clearance. Moreover, nanomedicines could also pass the epithelial barrier with the assistance of the designed carriers, which achieve rapid drug absorption via carrier‐mediated transports expressed in the lung, especially for biological drugs. However, for the local target medicine, high efficiency of circulation transportation or macrophage clearance could reduce the local drug enrichment, followed by reduced therapeutic efficiency.^[^ ^50^ ^]^ Therefore, researchers need to design drug delivery systems to improve lung retention or have the ability of epithelial‐targeted intracellular release.

### 2.4 Structure of Nucleic Acid

Not only do the biological characteristics affect the pulmonary bioavailability of the inhaled nucleic acid, but the specific structure of nucleic acid also causes their low absorption, especially for the RNAs. Unlike DNA, RNA is often a single‐strand structure, which is more fragile to break down.^[^ ^51^ ^]^ Moreover, the RNA backbone comprises alternating phosphate groups and sugar ribose, rather than deoxyribose for DNA, which has a free hydroxyl in the 2′ position. Under the aqueous condition, the free hydroxyl is deprotonated and attacks the phosphorus to form a transition state where the phosphorus is bonded to five oxygen atoms, followed by breaking the sugar‐phosphate backbone and cleaving the RNA chain. In addition, the airway also contains a small amount of nuclease from lysate epithelial cells that could direct the nucleic acid at a specific site. Therefore, the successful delivery of RNA‐based nucleic acid is still a challenge that blocks the breakthroughs in gene therapy. Improving the RNA stability via structure modification such as m6A and s2U decoration or encapsulation by nanocarriers to isolate from the aqueous environment and nuclease has been considered a potential assay to overcome this obstacle.^[^ ^52^ ^]^

### 2.5 Characteristics of Formulated Nanomedicine

As the nucleic acids are not stable as small molecules, encapsulation by the designed nanocarrier was the popular way for their pulmonary delivery. However, the characteristics of gene‐loaded platforms could also affect the pulmonary bioavailability of nucleic acids via the influence of their pulmonary deposition, mucus penetration, and cellular transportation behaviors, which are strongly related to the inhaled particle size, surface charge, and particle shape.

The size is the primary parameter that affects the pulmonary deposition of the drug‐loaded delivery platform.^[^ ^53^ ^]^ Inertial deposition is the major mechanism of therapeutic nucleic acid‐loaded nanocarriers in the airway with an aerodynamic diameter larger than 5 µm. The particles inhaled with large aerodynamic diameters exhibit inertial motion when the airway bifurcates, followed by impact and adhesion on airway walls at those bifurcation sites. The increased particle size and flow velocity enhanced particle deposition, mainly in the throat and upper bronchus. For the particle aerodynamic diameter between 2 to 8 µm, gravitational deposition was another major mechanism for their pulmonary deposition based on gravity overcoming the buoyant force and inducing particle adhesion in the bronchi or bronchioles. Whereas for the particles with aerodynamic diameters lower than 0.5 µm, their deposition mainly happens in the bronchiole and alveolus based on the movement pattern of Brownian motion. The reduced aerodynamic diameter enhanced their Brownian motion, which increased their chances of adherent in epithelial cells. Moreover, the size of nanocarriers also affects their mucus penetration and macrophage escapes, which has been described in the front section, suggesting designing a size‐changeable nuclide acid delivery system could improve their pulmonary bioavailability.

The shapes of inhaled particles also affect the pulmonary deposition by interception assay.^[^ ^54^ ^]^ The non‐spheric particles, such as fibers, could easily touch the airway walls and be trapped by the mucus layer. The fiber length significantly induced the fiber deposition due to interception in upper airways and reduced their effective total pulmonary accumulation. Moreover, research has confirmed that the fiber exhibited lower effective deposition in deeper airways than spherical particles with equivalent spherical diameters, suggesting the spherical carrier is more suitable for pulmonary drug delivery. In addition, the fiber‐shaped particles find it challenging to penetrate low‐viscosity holes and easily be trapped by the mucin proteoglycans. The alveolar macrophage phagocytosis is also changeable when targeting clearance fiber‐shaped particles. It's been proved that the phagocytosis process could happen when the fiber axis side is first trapped by the alveolar macrophage, while phagocytosis escapes when the fiber body side is touched.

The surface charge also affects the deposition, mucus penetration, and lysosomal escape process, which needs to be paid more attention during nucleic acid carrier development.^[^ ^55^, ^56^ ^]^ Compared to other deposition mechanisms, electrostatic precipitation only exhibits limited influence on particle pulmonary deposition. Even though most respiratory tracts were neutral, some surface areas still exhibited polarity charges and may induce electrostatic precipitation of particles with opposite charges. For the micro‐sized particles, the dispersion process causes friction between particles and particles to the inhalers, which may affect their deposition effects. A researcher has found that charged particles deposited in the alveolar region could be five times higher than those in the tracheobronchial region.^[^ ^57^ ^]^ In addition, the deposited nucleic acid carrier needs to cross the mucus layer and avoid electrostatic adsorption by the mucin proteoglycans and alveolar surfactant. The carrier with a positive surface charge could be trapped in mucus, reducing its pulmonary bioavailability.

## 3 Materials Development for Nucleic Acid Pulmonary Delivery

**Figure 3:**
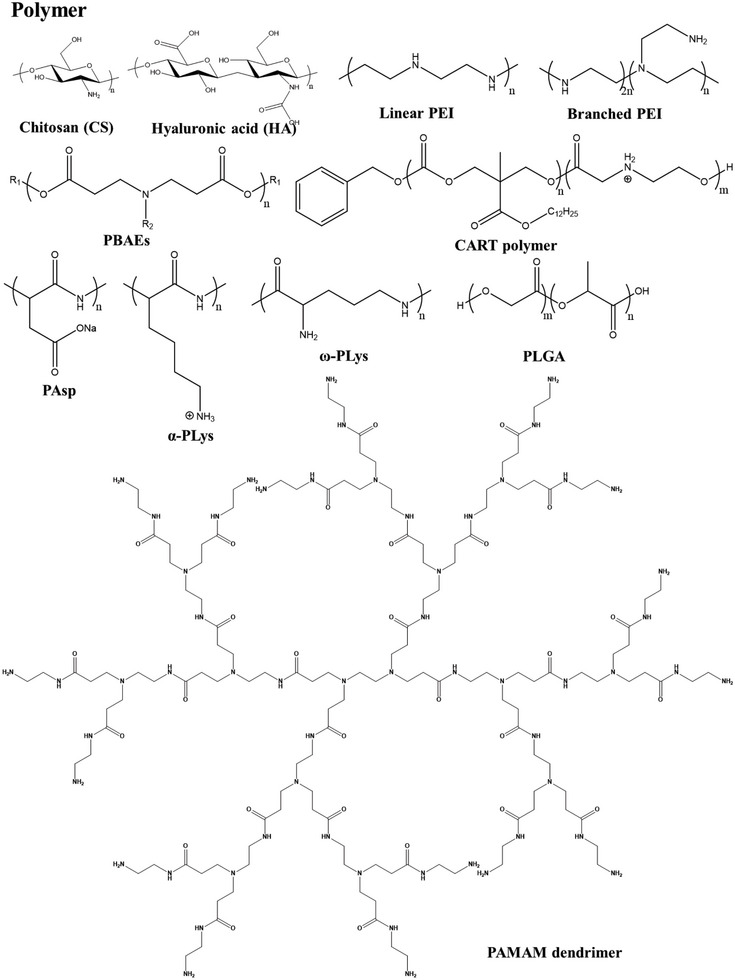
Chemical structures of commonly used polymeric materials as nanocarriers for pulmonary gene delivery.

For gene‐effective pulmonary delivery, materials design needs to meet the specific requirement of high nucleic acid affinity, easy biodegradation, and high safety. Typically, positively charged materials are essential for gene loading via electrostatic adsorption. Nucleic acids that carry negative charges could bind with positively charged materials to form gene‐carrier complexes or be encapsulated into the core of nanoparticles. The design and development of novel materials have been considered the sticking point for nucleic acid formulation and pulmonary delivery (**Figure** **3**).

### 3.1 Polymeric Materials

#### 3.1.1 Natural Polysaccharide

Polysaccharides are a series of macromolecules composed of monosaccharide units linked by glycosidic bonds in specific regular order. As natural products, most polysaccharides have been considered biodegradable and have been explored for their applications as drug delivery carriers. Several natural polysaccharides that carry charges in a neutral environment have been investigated as potential gene carriers for effective delivery.

Chitosan was a natural polysaccharide forming with β‐(1→4) glycosidic bonds‐linked D‐glucosamine and N‐acetyl‐D‐glucosamine in random order, produced from the deacetylated chitin which is the major component of shells of crustaceans. Chitosan has been investigated and is considered to have good biocompatibility. The deacetylation leads to the exposure of free primary amino groups in chitosan with the pKa ≈6.5, which would carry positive charges in an acid environment and bind with therapeutic genes to form the nanocomplexes.^[^ ^58^ ^]^ One research group has prepared a PEGylated chitosan‐based DPI system for CRISPR‐Cas9 pulmonary delivery and achieved high stability and transfection efficiency.^[^ ^59^ ^]^ Moreover, chitosan derivatives such as trimethyl chitosan and carboxymethyl chitosan were also designed and investigated for nucleic acid pulmonary delivery.^[^ ^60^, ^61^ ^]^ The survivin siRNA was encapsulated by the baclofen functionalized trimethyl chitosan with particle size ≈220 nm and inhaled via a pressurized metered dose inhaler (pMDI). The formulation's fine particle fraction (FPF) reached 45.39 ± 2.99%, suggesting chitosan derivatives could deliver therapeutic genes into deep lungs. More than could be used for gene binding directly, another research group has also reported using sprayed dry chitosan as a dispersion enhancer to improve the lung deposition of pDNA‐loaded lactose‐LPD (lipid:polycation:pDNA) particles, which reduced the aggregation during the DPI aerosolization.^[^ ^62^ ^]^

Hyaluronic acid (HA) is an anionic linear non‐sulfated glycosaminoglycan with units of D‐glucuronic acid and N‐acetyl glucosamine and with a large molecular size that is synthesized in the plasma membrane. The HA is a component of our extracellular matrix and exists in connective tissue and epithelial tissue, with excellent biodegradability but no immunogenicity. Due to containing free carboxyl groups, HA usually expresses negative charges and cannot directly generate electrostatic adsorption with nucleic acid. Most HA applications in gene delivery were used as a shield to neutralize the excessive positive charges and reduce mucosa stimulation. A research group developed HA‐coated liposome‐protamine‐siRNA complexes for Bcl‐2 siRNA pulmonary delivery.^[^ ^63^ ^]^ With various amounts of HA coating on the surface, charges of the carrier‐siRNA complex were changed from positive (40 mV) to negative (‐40 mV), which not only reduced the cytotoxicity of the cationic carrier but also improved gene silencing efficiency.^[^ ^64^ ^]^ In addition, another research group reported that HA alone spray‐freeze‐drying with pDNA could be a potential assay for the therapeutic gene's pulmonary delivery.^[^ ^65^ ^]^ Authors prepared pDNA encapsulated HA porous microparticles with a size of ≈5–10 µm, which significantly improved the stability of pDNA for 12 months under dry conditions with FPF ≈25%, suggesting this HA microparticle could be an effective delivery carrier for nucleic acid pulmonary delivery.

#### 3.1.2 Polyethylenimine (PEI)

Cationic polymers are a series of non‐viral vectors being investigated for effective therapeutic gene delivery. PEI is the most classic cationic polymer being investigated for gene delivery in vitro. The PEIs were formed by repeated units of ethylamine, which contain large amounts of amino groups. Linear PEI only has secondary amino groups, while primary and tertiary amino groups are also included in the branched PEI. Under physiological pH, amino groups in PEI ionized and contained positive charges to combine the negatively charged nuclei acid and form the nanocomplexes, effectively improving nucleic acid stability and transfection.

Recently, transferrin (Tf) modified PEI was synthesized to target delivery of GATA3 siRNA to activated T cells.^[^ ^66^, ^67^ ^]^ The researcher confirmed the silencing efficiency of GATA3 over 88% in human precision‐cut lung slices ex vivo after co‐incubation with siRNA‐combined Tf‐Mel‐PEI polyplexes. Another research group constructed a MAL‐PEG‐PEI complex to combine siRNA (RUNX1 and Gli1) to form micelles followed by conjugate anti‐Sca1 on the surface to target lung‐resident mesenchymal stem (LR‐MSCs).^[^ ^68^ ^]^ The siRNA‐loaded micelles with particle size ≈40 nm and charges of 10 mV could effectively transfect into LR‐MSCs and silence targeted genes in vitro and also exhibited therapeutic efficiency against pulmonary fibrosis in C57BL/6 mouse pulmonary fibrosis model via intravenously injected.

Even though various PEI‐based carriers have been investigated for nucleic acid delivery in vitro for pulmonary local disease treatment, it seems the physiological barrier still needs to be overcome before expanding their application directly in pulmonary delivery. A PEI (M_w_ = 25 kDa) and nucleic acids nanocomplex encapsulated microparticles have been prepared via spray drying nanocomplex with trehalose.^[^ ^69^ ^]^ These microparticles with a mass median aerodynamic diameter (MMAD) of 3.17 µm and spherical morphology improved their pulmonary deposition efficiency. The spray drying process did not reduce the transfection efficiency, suggesting that PEI‐based nanocomplex could be a potential nucleic acid delivery carrier for DPI preparation. Another research group introduced a layer‐by‐layer polymer prepared with chitosan, dextran sulfate, and mannose‐g‐PEI for miRNA‐34a pulmonary delivery via preparing DPI with chitosan, leucine, and mannitol as a multiple‐excipients.^[^ ^70^ ^]^ The DPI exhibits good pulmonary deposition with MMAD ≈5.8 µm and high ability cellular uptake. Although the application of PEI in inhalation demonstrated advances in gene delivery, the high chances of irritation due to their strongly positive charges still restrict its application. A suitable modification of PEI structure or formulation to shield the extra charges should be the development direction in the future for their clinical application.

#### 3.1.3 Polyamidoamine (PAMAM) Dendrimers

Polyamidoamine (PAMAM) dendrimers are a type of hyperbranched polymer. The PAMAM is constructed starting with an ethylenediamine core, followed by conjugation of repetitive branching amidoamine and a primary amine terminal surface. The size and molecular weight of PAMAM dendrimers are related to the times of generation process from the central core and could form spherical particles after generation 4. The surface primary amino groups carry positive charges and could directly bind with a nucleic acid to form the nanoparticles. At the same time, the internal secondary and tertiary amino groups could also be ionized under an acid pH environment and exhibit the proton sponge effect, helping particles escape from the lysosomal degradation and nucleic acid cytoplasm release. Similar to the branched PEI, the PAMAM also faces the problem of irritation due to its high density of positive charges under physiological conditions, which limits its application in vivo or clinical investigation.

A research group prepared TNF‐α siRNA‐loaded PAMAM dendrimer (generation 3) to treat acute lung inflammation.^[^ ^71^ ^]^ The siRNA‐loaded dendrimer exhibited strong siRNA condensation and cellular uptake by the macrophages. Via intranasal administration in vivo, the siRNA‐loaded dendrimer could significantly reduce the TNF‐α generation and prevent LPS‐induced inflammation in the pulmonary. Another research group synthesized (3‐carboxypropyl) triphenylphosphonium bromide (TPP) modified PAMAM (generation 4) for EGFP siRNA condensation.^[^ ^72^ ^]^ With the optimization of surface TPP density, the cellular uptake and targeting gene silencing was highest at around 45%. Moreover, the siRNA‐loaded 12TPP‐PAMAM dendrimer was spray‐dried with mannitol to prepare DPI and dispersed with HFA 227 to prepare pMDI, respectively. The DPI and pMDI exhibit FPF of 39% and 50%, respectively, suggesting both formulations were suitable for siRNA pulmonary delivery. The application of PAMAM is not limited to small nucleic acid delivery. One research group has prepared a series of PAMAM derivatives for Cy5‐EGFP mRNA condensation.^[^ ^73^ ^]^ The PAMAM dendrimers with suitable amounts of p‐toluenesulfonyl groups and imidazole groups modification could successfully load mRNA and improve their endosomal escape, which exhibited better cellular transfection than the commercial jetPEI. Based on the investigation described above, the PAMAM dendrimers could be considered the potential gene carriers for their pulmonary delivery.

#### 3.1.4 Poly (β‐Amino Esters) (PBAEs)

Poly(β‐amino esters) (PBAEs) is a series of cationic polymers with tertiary amine groups that could electrostatically interact with nucleic acid with negative charges. The backbones of PBAEs contain degradable ester bonds that provide excellent biocompatibility and biodegradability. The PBAEs are synthesized by a one‐pot assay of amines to acrylates, thus forming diverse structures due to the combination of different monomers, leading to the multi‐function of PBAEs. Moreover, PBAEs could conjugate with other polymers or formulate with other materials to achieve the desired delivery ability. Therefore, PBAE polymer libraries have been designed and investigated for their applications in nucleic acid effective and safe delivery, including in the pulmonary route.^[^ ^74^ ^]^

Mastorakos et al. designed a PBAE modified with a high density of PEG corona on the surface for pulmonary gene delivery.^[^ ^75^ ^]^ The high density of hydrophilic PEG corona improved the PBAEs‐based nanoparticles’ mucus‐penetrating ability. The Cy3‐labeled plasmid DNA‐loaded PBAE nanoparticles exhibited highly uniform airway distribution, reduced particle aggregation, and inhibited alveolar macrophage phagocytosis after intratracheal administration in BALB/C mice. The signal dose of pulmonary delivery of DNA‐loaded PEAE‐based nanoparticles achieved robust transgene expression with lower toxicity of airway tissues compared to PEI‐based carriers. Another research group prepared PBAE/ guanidinylated O‐carboxymethyl chitosan (OCMCS) nanoparticles for survivin siRNA pulmonary delivery via nebulization. The inhaled PBAE/siRNA/FITC‐GOCMCS particles exhibited an FPF of 57.8 ± 2.6% with a strong siRNA silence ability in bronchial epithelial cells, suggesting this delivery system was suitable for siRNA pulmonary delivery. The hyperbranched PBAEs were also used to encapsulate mRNA and prepare a nebulizer for pulmonary inhalation.^[^ ^21^ ^]^ The hyperbranched PBAEs exhibited stable particle size at high mRNA binding doses, while the linear PBAEs formulated particles were aggregated into large particles with particle sizes over 1000 nm. The signal dose of nebulizer inhalation transfected 24.6% of pulmonary epithelial cells, illustrating the hyperbranched PBAEs as the potential mRNA carriers. Rotolo et al. constructed a polymer library of 166 PBAE polymers with various types of branching structures, linkers, and end capping, and the P76 could effectively deliver mRNA and crRNA via preparing as a nebulizer for SARS‐CoV‐2 prevention.^[^ ^76^ ^]^ The P76 of PBAEs could effectively deliver genes to pulmonary from different animal species and exhibited increased expression of targeted proteins with low toxicity, indicating the PBAE‐based polymeric could be a potential therapeutic RNA carrier for pulmonary administration.

#### 3.1.5 Charge‐Altering Releasable Transporter (CART)

The charge‐altering releasable transporters are a series of amphiphilic polycations with a lipophilic block modified with various lipid side chains and a cationic block for nucleic acid binding. The cationic blocks of CART are similar to the PBAEs, which form by the degradable poly(α‐amino ester) backbone.^[^ ^77^ ^]^ The secondary amino groups exhibit positive charges at physiological pH and binding nucleic acid. Under the acid endosomes environment, these CART polymers could be self‐degraded into the neutral, non‐toxic diketopiperazine and achieve the loaded gene rapid release into the cytoplasm, which avoids the biocompatibility issues caused by the undegradable cationic polymers.

The CART system was designed by the research groups of Dr. Wender and Dr. Waymouth, which are being used for mRNA delivery. The optimized CART polymer binary mixtures could significantly increase the mRNA translation efficiency to 80% in lymphocytes in vitro compared to the commercial Lipofectamine 2000 with that of 12%.^[^ ^77^ ^]^ For SARS‐CoV‐2 prevention, CARTs are also used for receptor binding domain (RBD) mRNA loading and intramuscular vaccination. The vaccinated mice generated RBD‐specific neutralizing antibodies in both circulation and bronchial fluids, suggesting the formation of cellular and humoral immunity.^[^ ^78^ ^]^ Based on the structure of the first‐generation CART polymer, they designed an oligo(serine ester) based secondary generation of CART polymer, which could be degraded into neutral oligo(seine amides) and diketopiperazine.^[^ ^79^ ^]^ The research data exhibited that these CARTs were stable at a preparation pH of 5.5 while starting degradation at pH 7.4. After intramuscular injection in vivo for 7 h, the Fluc mRNA‐loaded ser‐CART showed a strong bioluminescence expression. The intravenous injection resulted in luciferase expression primarily located in the spleen, suggesting their lymphocyte targeting ability. Moreover, lysine‐derived CART is also being investigated for mRNA lung‐targeted delivery.^[^ ^80^ ^]^ Without other targeting ligands, lysine‐based CART exhibited lung accumulation after intravenous injection, suggesting their potential application in pulmonary RNA delivery.

#### 3.1.6 Poly(Amino Acid)

Amino acids such as lysine contain more than one amino group, and lysine‐based poly(amino acid) has also been considered potential cargo for therapeutic gene delivery. The free primary amino groups in poly‐lysine carry positive charges under a physiological environment and could bind with negatively charged nucleic acid for their effective delivery. The research groups of Kaminskas investigated the application of lysine‐based poly(amino acid) in drug pulmonary delivery. They synthesized generation 4 PEGylated polylysine dendrimers with different lengths of PEG chains with different molecular weights. The absorption of PEGylated polylysine dendrimers was negatively related to their molecular weight, and the polylysine dendrimer modified by PEG_2300_ exhibited longer retention time in pulmonary and bronchoalveolar lavage fluid (BALF) after administrated to the lungs as a liquid instillation.^[^ ^81^ ^]^ Besides the polylysine, poly(aspartic acid)‐based polymeric nanoparticles are also being investigated for mRNA delivery locally or systemically. Intravenous injection of PEGylated poly(aspartic acid) could achieve pulmonary targeted mRNA transfection with increased cell viability compared to non‐PEGylated nanoparticles.^[^ ^82^ ^]^ This PEGylated poly(aspartic acid) nanoparticle could be a potential lung‐targeted therapy for the local disease.

Another type of cationic polypeptide was designed for nucleic acid binding and successful delivery. A PEG‐modified cell‐penetrating peptide was designed with octa‐arginine (R8) and a peptide derived from fibroblast growth factor 2 and KL15 sequence for the gene pulmonary delivery.^[^ ^83^ ^]^ The pDNA was effectively combined with polypeptide chains, and the PEG modification shielded the positive charges of nanoparticles. PEGylation over 40% helped the particle to be stable in the BALF and further rapidly diffused in the mucus in vitro. The designed nanoparticle reserved a high transfection activity of nucleic acid in human bronchial epithelial cell lines and precision‐cut lung slices in vitro and exhibited improved transfection in vivo compared to PEI‐based carriers. Qiu et al. reported KL4 peptide‐based materials for nucleic acid delivery in vitro and in vivo. The KL4 peptide is a cationic peptide with repeating KLLLL sequences that could combine with siRNA for lung cancer cells and bronchial epithelial cells effectively transfection.^[^ ^84^ ^]^ They further modified the KL4 peptide with a 12‐unit PEG for the mRNA encapsulation and pulmonary delivery. PEG‐KL4 encapsulated with mRNA at a ratio of 10:1 exhibited the most effective epithelial cell transfection. After spray drying or spray freeze‐drying with mannitol, Fluc‐mRNA‐loaded dry powder inhalers were produced and showed effective luciferase expression in the deep lung in mice after intratracheal administration for 24 h.^[^ ^85^ ^]^ The same research group also designed a series of pH‐responsive peptides containing several histidines or 2,3‐diaminopropionic acid residues.^[^ ^86^, ^87^ ^]^ The plasmid DNA could form a complex with the peptides due to electrostatic adsorption, and the peptides could further be ionized in an acid lysosome environment and disrupt the lipid membrane, followed by the release of nucleic acid into the cytoplasm. The DPIs were prepared with mannitol via spray drying or spray freeze drying, followed by an investigation of aerosol desperation and transfection in vivo, exhibiting an FPF over 50% and better transfection efficiency compared to lipofectamine 2000.

Except for the artificial poly(amino acid) or peptide as the carrier for gene pulmonary delivery, some natural peptides and proteins could also be investigated to transport therapeutic nucleic acid into the lung effectively. Gelatin is a mixture of peptides and proteins from collagen hydrolyzation. Due to their biodegradable ability and multi‐functional groups, gelatin is also considered an inhalation carrier for small molecules or nucleic acid pulmonary administration. Cationized gelatin nanoparticles were used to load Cytosine‐Phosphate‐Guanine‐Oligodeoxynucleotides (CpG‐ODN) as an immune adjuvant to improve immunotherapy efficiency on hypersensitivity in horses.^[^ ^88^ ^]^ The gelatin nanoparticles were administered via nebulization with an FPF over 65%, maintaining the IL‐10 stimulated release both in vitro and in vivo. The gelatin could also be used for the drug's pulmonary administration via preparing DPIs. The gelatin‐based nanoparticles could be spray‐dried with leucine to form a microparticle with MMAD of 2.59 ± 0.31 µm and a respirable fraction of around 50%, exhibiting good aerosolization properties enabling effective deposition in the airways.^[^ ^89^ ^]^

Albumin is another type of natural peptide being investigated as a therapeutic gene carrier for pulmonary disease treatment. Albumin, as a component of epithelial and endothelial cells, exhibits properties of biodegradability and biocompatibility, suggesting a safety nanocarrier for gene delivery. A bovine serum albumin‐based nanoparticle was prepared to deliver siRNA for KRAS mutant lung cancer treatment in vitro.^[^ ^90^ ^]^ The siRNA‐loaded albumin nanoparticles with a size of less than 200 nm could be effectively uptaken by A549 cells and achieve KARS knockdown. Moreover, human serum albumin is also being applied as a dispersion enhancer to improve the DPI aerosol performance. Chow et al. optimized the spray‐dried parameters and formulation to prepare siRNA‐loaded mannitol dry powders with albumin incorporation.^[^ ^91^ ^]^ The albumin enriched on the particles' surface modified the morphology and dispersion of microparticles with FPF over 50%. The albumin also protected the bioactivity of siRNA with a gene silence ratio of ≈78% after the spray‐drying process.

#### 3.1.7 Polycarbonate

Polycarbonate is a series of polymers with biocompatibility and degradability that has been investigated in formulating drug delivery systems to achieve prolonged drug release. Block copolymers formed by various types of monomers were developed, some of which have been approved by the Food and Drug Administration (FDA) for clinical application. Poly(lactide‐co‐glycolide) (PLGA) was one of the most investigated polymers widely used in pulmonary inhalation development. Unlike other cationic materials, PLGA will not bind nucleic acid with electrostatic adsorption due to a lack of amino groups. PLGA mainly works as package materials that encapsulate therapeutic genes inside the core of nanoparticles to improve the stability of genes. To prepare the formulation, gene contained buffer was mixed with PLGA dissolved organic solvent followed by sonicating to form w/o emulsion. Then, the emulsion was added to the aqueous solution and formed w/o/w double emulsion via sonicating. Finally, the organic solvent is evaporated to obtain gene‐loaded nanoparticles. Dalirfardouei et al. reported a DNA vaccine (pcDNA3.1‐Mtb72F) encapsulating plasmid in PLGA nanoparticles to improve immunogenicity and prevent TB infection.^[^ ^92^ ^]^ The result suggested that PLGA particles with a size smaller than 200 nm can effectively induce cellular immunity and improve interferon‐γ and interleukin‐4 generation, indicating the PLGA nano vaccine is a promising immune adjuvant. Moreover, another research group reported a cell‐penetrating peptide‐coated pDNA‐loaded PLGA nanoparticle for lung delivery. The formulation successfully encapsulated pDNA and achieved prolonged release.^[^ ^93^ ^]^ Cell‐penetrating peptide (CPP) modification of PLGA nanoparticles improved their cellular uptake in BEAS‐2B and A549 cells. It provided an endosomal escape ability that could effectively enhance targeted gene expression, suggesting a promising gene delivery system into the lung.

### 3.2 Lipid Materials

**Figure 4:**
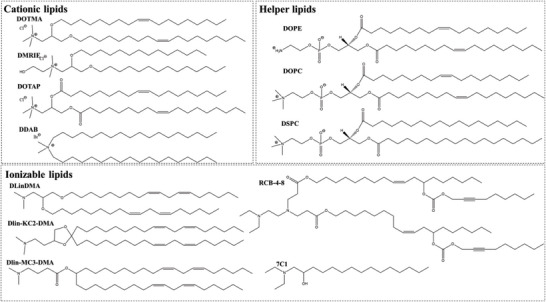
Chemical structures of commonly used lipid materials as nanocarriers for pulmonary gene delivery.

Lipid materials have been widely investigated in drug delivery system development and have attracted considerable interest due to their endogenous source and excellent safety. Pulmonary surfactants, as the major component maintaining surface tension, contain many types of lipids such as phospholipids, triglycerides, cholesterol, and fatty acids. As endogenous functional components, lipid‐based materials, therefore, are more promising for pulmonary formulation designs (**Figure** **4**).

#### 3.2.1 Cationic Lipids

Cationic lipids are a series of amphiphilic compounds consisting of a cationic headgroup conjugate with one or more hydrophobic fatty acid tails with different linkers. The cationic headgroups always contain one or more prominent amino groups such as quaternary ammonium salts, charged other amino groups, guanidine, or heterocyclic compounds, which lead the whole compounds to carry positive charges at physiological pH. The cationic headgroups of cationic lipids absorbed nucleic acid via electrostatic adsorption, and its amphiphilic structure would help the liposome or lipid nanoparticle preparation for therapeutic gene delivery. The structure of lipid materials, such as the headgroup numbers, hydrophobic lipid chains, and their structure (unsaturation degree), are the main factors that affect the gene loading efficiency, formulation stability, cellular uptakes, and endosome escape.

N‐[1‐(2,3‐dioleoyloxy)propyl]‐N,N,N‐trimethylammonium chloride (DOTMA) as a type of tetra‐methylated di‐octadecyl‐trimethylammonium propane analog, has been considered a cationic lipid and being used for gene delivery vector formulation. The DOTMA contains a quaternary ammonium headgroup and two unsaturated lipid chains linked through ether bonds. Combined with other essential materials, DOTMA has been investigated as a major component for therapeutic nucleic acid pulmonary administration. Kurosaki et al. designed a TGF‐β shRNA encoded pDNA loaded lipopolyplex for idiopathic pulmonary fibrosis (IPF) treatment.^[^ ^94^ ^]^ They optimized a formulation of lipopolyplex containing pDNA, poly‐Lysine, DOTMA, and N‐lauroylsarcosine (LS) with a charge ratio of 1:2:2:4, and the prepared formulation exhibited the highest TGF‐β shRNA expression, which effectively inhibited IPF progression via intravenous administration. Another research group prepared a double layer of lipid nanoparticles (LNPs) to load the pDNA‐PEI complex with DOPE/STR‐R8 inner lipid core and DOTMA/YSK05 outer lipid.^[^ ^95^ ^]^ The GALA peptide decoration provides the LNPs targeting ability to the lung endothelium and endosomal escape ability. After formulation optimization, the transfection activity of double‐layer LNPs was significantly improved, observed both in vitro and in vivo.

DMRIE (1,2‐dimyristyloxy‐propyl‐3‐dimethyl‐hydroxy ethyl ammonium bromide) is another cationic lipid material derived from DOTMA. Johler et al. compared the transfection efficiency of mRNA in human bronchial epithelial cell lines using differently formulated liposomes (Lipo2000 or DMRIE‐cholesterol) and PEI‐based polyplexes in vitro.^[^ ^96^ ^]^ The result suggested that mRNA formulation prepared with cationic materials improved their resistance to shear stress during the nebulization process, which exhibited no significant effects for mRNA‐loaded polyplexes but slightly reduced the transfection efficiency of lipoplexes and liposomes. The DRMIE‐cholesterol liposomes showed higher mRNA transfection efficiency than PEI‐based polyplexes but still not effectively like the lipo2000 as vectors, which might be due to the affinity between genes and cationic materials, suggesting a deep investigation needs to be done to optimize the balance between nucleic acid loading efficiency and intracellular release.

DOTAP exhibits a similar structure to the DOTMA, which both contain quaternary ammonium headgroup and two unsaturated lipid chains, only exhibiting difference by using an ester bond to replace the ether bonds to link those parts. DOTAP could also be directly binding with negatively charged genes and applied as a major component of a lipid‐based delivery system for their pulmonary delivery. Thanki et al. reported a lipid‐polymer hybrid nanoparticles (LPNs) drug delivery system helps siRNA pulmonary delivery.^[^ ^97^ ^]^ The LPNs contained cationic lipidoid 5 or DOTAP as a cationic lipid coating layer and a PLGA nanoparticle core. The loaded siRNA was present as complexes with the cationic lipid and located near the LNPs surface. Lipidoid 5 and DOTAP‐modified LPNs all improved siRNA pulmonary retention after pulmonary administration, suggesting a promising siRNA delivery cargo for pulmonary disease treatment. Another research group prepared solid lipid nanoparticles (SLNs) using lecithin, cholesterol, and PEG‐hydrazone‐C18 (PHC) to encapsulate siRNA/DOTAP complexes for gene pulmonary inhalation.^[^ ^98^ ^]^ The SLN dry powder was prepared via various dry methods such as spray drying, conventional shelf freeze‐drying, and thin‐film freeze‐drying assay (TFFD) with mannitol as excipients. The in vitro aerosol performance results suggested that TFFD exhibited the best in vitro deposition behavior with FPF over 37% and MMAD of 3.96 ± 0.97 µm, indicating TFFD could be a promising drying method to prepare inhalable LNP dry powder.

Dioctadecyldimethylammonium bromide (DDAB) is another type of cationic lipid investigated for systemic or local delivery of therapeutic agents. In the structure of DDAB, two saturated hydrophobic lipid chains are directly connected with the quaternary ammonium headgroup without linkers, representing a polar environment for gene binding but with lower fluidity. Hattori et al. reported a siRNA lipoplexes system for their targeted delivery to the lung via intravenous injection.^[^ ^99^ ^]^ The cationic lipid combined with helper lipid and cholesterol to prepare cationic liposome and siRNA lipoplexes were formulated via mixing the siRNA and liposomes. The siRNA lung targeted delivery efficiency was strongly affected by the length of cationic lipid chains and the helper lipid composition, which exhibited DDAB‐based cationic liposome as a potential delivery system for pulmonary gene delivery. Moreover, the cellular uptake behaviors of DDAB‐based liposomes in pulmonary alveolar macrophages and lung epithelial cells were also investigated in vitro to simulate their destination after inhaling.^[^ ^100^ ^]^ The data suggested that protein corona in BALF did not affect the uptake of DDAB‐based liposomes in pulmonary epithelial cells. However, in macrophages, the protein corona could induce the rapid phagocytosis of liposomes followed by exocytosis. The influence of protein corona in cellular uptake may guide the future gene delivery systems design and help improve their transfection efficiency in the pulmonary delivery route.

#### 3.2.2 Ionizable Lipids

Ionizeable lipids are a series of lipid materials derived from the traditional cationic lipids via replacing the quaternary amino groups with ionizable groups such as a tertiary amine. Due to their unique physicochemical feature, ionizable lipids need to be charged first to encapsulate nucleic acid during the LNP formulating process. Unlike the permanently charged cationic lipids, ionizable lipids exhibit a neutral surface at a physiological pH environment and only carry positive charges under an acid environment, which avoids irritation in the airway after inhalation and improves mucus penetration ability. After cellular uptake, the acid endosome environment induces the ionizable lipid to be positively charged, followed by disrupting the endosome membranes via electrostatic interactions and releasing nucleic acid into the cytoplasm. The structure of ionizable lipids also includes hydrophobic lipid chains, ionizable headgroups, and linkers between them. Similar to the cationic lipids, the ionizable headgroups and the structure of lipid chains (unsaturation degree and length) are the main factors that affect formulation properties and their transfection efficiency. The pKa of designed ionizable lipids should be low enough to keep most of them unprotonated during circulation but could rapidly be charged in the endosome's acid pH environment.

Various ionizable lipid materials were designed and synthesized for LNP preparation and therapeutic gene delivery. The Dlin‐DMA lipid family was the most investigated ionizable lipid, including Dlin‐DMA and their derivatives Dlin‐MC3‐DMA (MC3) and Dlin‐KC2‐DMA (KC2). The Dlin‐DMA lipid family consisted of an amine head group and dual linoleic tails connected by different linkers. Replacing the ether linkage with a ketal group could improve the transfection efficiency, while replacing it with an ester group exhibited the opposite effects.^[^ ^101^ ^]^ Therefore, the Dlin‐KC2‐DMA was developed with a pK_a_ of 6.7 and improved siRNA silence efficiency. The length of the carbon chain in the amino headgroup also affects the features of lipids, and the increased chain length could improve the pK_a_ of lipids. The MC3, with a pK_a_ of 6.44, was generated by prolonging the headgroup lipid chain and replacing the linker with an ester group. The siRNA transfection efficiency of MC3 was significantly improved compared to Dlin‐DMA. Zhang et al. reported siRNA‐loaded LNPs for asthma treatment, prepared using MC3, DOPC, peptide conjugated cholesterol, and PEG‐DMG (Airway epithelial cell‐specific delivery of lipid nanoparticles loading siRNA for asthma treatment). The cyclic peptide as a rhinovirus capsid protein analog provides the LNPs airway epithelial cells (ACEs) targeting ability and the significantly improved gene silence efficiency in OVA‐challenged asthmatic mice via intratracheal microspray. Another research also uses MC3 as an ionizable lipid to prepare LNPs for successful mRNA pulmonary delivery and transfection. Kim et al. prepared the LNPs with MC3, cholesterol analog, DSPC, and DMG‐PEG_2K_ for fibrosis transmembrane conductance regulator (CFTR) encoding mRNA pulmonary transfection.^[^ ^14^ ^]^ Optimized LNPs could rapidly cross mucus with endosome escape ability, which achieves high transfection efficiency in a CFTR‐deficient animal model via pulmonary administration. Based on the related investigation, the Dlin‐DMA lipid family has been considered a promising gene vector for nucleic acid pulmonary administration.

Based on the success of the Dlin‐DMA lipid family in gene delivery, more ionizable lipids have been designed, and LNPs were further formulated to achieve nucleic acid delivery. Most of them were only investigated in circulation gene delivery with excellent transfection efficiency, and some surprisingly exhibit lung targeting ability. Qiu et al. reported a series of ionizable lipids and investigated the effect of different amine headgroups and acrylamide hydrophobic tails in plasma LNPs protein corona formation and their organ targeting efficiency.^[^ ^4^ ^]^ The prepared lipids with an amide bond linker could achieve pulmonary accumulation, while those with an ester bond linker only targeted the liver.

The research group of Dr. Daniel Anderson reported a novel C15 epoxide‐modified low‐molecular‐weight polyethyleneimine (7C1) exhibited effective non‐liver siRNA accumulation via systemic delivery.^[^ ^102^ ^]^ 7C1 lipid preferentially transfected endothelial cells in multiple organs with the highest gene silencing efficiency in the lung. Based on the novel discovery, they further investigated the potential of 7C1‐based LNPs in pulmonary gene delivery.^[^ ^103^ ^]^ By optimizing the formula compositions, such as the structure of ionizable lipids, molar ratio of helper lipids, and surface PEG density, they identified potential LNP formulations for low‐dose messenger RNA pulmonary via a nebulization. Inhaled mRNA encoding neutralizing antibody targeting haemagglutinin loaded in 7C1‐based LNPs could effectively protect the mice from infection of the HIN1 subtype of influenza virus. Moreover, Anderson et al. recently reported their latest designed ionizable lipid for LNPs formulation and pulmonary delivery of CRISPR–Cas9 gene editors.^[^ ^104^ ^]^ They developed a reaction system to connect 72 different amino headgroups and 10 different aliphatic alcohols via nitro ricinoleic acrylate linker, which synthesized a lipid library containing 720 new ionizable lipids for further investigation on gene delivery. The transfection efficiency of synthesized ionizable lipids was investigated by preparing LNPs with helper lipids, cholesterol, and DMG‐PEG_2K_. The optimized RCB‐4‐8 LNPs were further intratracheal administrated, and their transfection efficiency was significantly improved compared to MC3 ionizable lipids in vivo, suggesting its other potential inhalable lipid materials for mRNA effective pulmonary delivery.

### 3.3 Extracellular Vesicles

Extracellular vesicles (EVs), also known as exosomes, are a series of exocytotic particles composed of phospholipid bilayers containing various lipids, proteins, and receptors on the surface and could encapsulate nucleic acids or other small molecules inside. The generated EVs with quorum sensing function are induced by intracellular signaling activation, which plays essential roles in inflammatory signaling transferring and genetic information sharing. With their excellent biosafety and targeting ability, EVs are also considered promising nucleic acid‐targeted delivery carriers, and their investigation in pulmonary disease treatment is underway.^[^ ^105^ ^]^ To achieve satisfactory targeting efficiency and prolonged retention time, EVs could be engineered via surface decorating and optimization, making bio‐engineered EVs more feasible in nanotechnology in the nucleic acid pulmonary delivery application.

Han et al. reported using a mice serum isolated EVs as a small RNA carrier for murine pulmonary inhalation using nebulization.^[^ ^106^ ^]^ The nebulizer with a vibrating mesh successfully atomized the EVs and achieved the targeted RNA delivery of the macrophages and epithelial cells. The siMyd88‐loaded EVs reduced the LPS‐induced inflammation and pulmonary injury in mice, suggesting the nebulization of EVs could be a potential route for small RNA pulmonary delivery. Dr. Ke Cheng's research group reported a series of research using specific types of exosomes in biomedicine and vaccine pulmonary delivery. They identified a series of adult lung cells with both epithelial and mesenchymal markers expression and exhibited a tissue‐repairing ability named lung spheroid cells (LSCs). The exosomes generated from LSCs were inhaled into fibrosis mice models with bleomycin or silica‐induced pulmonary injury.^[^ ^107^ ^]^ Inhalation of LSC‐EVs could reverse the pulmonary damage and fibrosis via reestablishing lung structure and inhibit the proliferation of myofibroblast, which exhibited a potential for pulmonary regeneration. They successfully promoted the expansion of the application of LSC‐EVs in pulmonary vaccine development. The SRAR‐CoV‐2 RBD conjugated LSC‐EVs were designed to induce the generation of RBD‐specific antibodies and mucosal immunization.^[^ ^108^ ^]^ After vaccination via nebulization, the RBD‐specific IgG and IgA antibodies were elicited, followed by the activation of T cells with a Th1‐like cytokine expression profile in mice. Moreover, the spike protein encoding mRNA‐loaded LSC‐EVs and liposomes were generated to evaluate the application of LSC‐EVs in vaccine development.^[^ ^109^ ^]^ To overcome the storage limitation of mRNA formulations, they freeze‐dried the mRNA‐loaded LSC‐EVs to prepare a dry powder vaccine that was kept at room temperature for up to 28 days. The DPI vaccine with LSC‐EVs as carrier improved the biodistribution in the lungs of rodents and nonhuman primates and elicited the immune responses with improved IgG and IgA secretion, suggesting the LSC‐EVs a superior gene carrier to the liposomes in pulmonary delivery.

### 3.4 Virus‐Based Carriers

Virus‐based delivery vectors have taken an important stage in gene therapy due to their evolutionary ability to infect host cells. Adeno‐associated viral (AAV), as a type of small single‐stranded DNA nonpathogenic parvovirus, exhibited less immunogenicity compared to adenovirus. The AAV‐based vectors have been evaluated in several clinical trials for nucleic acid transfection in treating Leber congenital amaurosis type 2 (LCA2) and spinal muscular atrophy (SMA). The application of AAV as vectors for pulmonary nucleic acid delivery has also been investigated to improve mucus penetration. The interaction of AAVs and bronchial mucus has been confirmed as the key reason for preventing virus penetration and host infection efficiency in the lung.^[^ ^110^ ^]^ The glycan receptors as the target of AAVs existed rich in bronchial mucus. The AAV binds with these “decoy” receptors and is trapped in the mucus layer, followed by clearance from the pulmonary airway. Duncan et al. evaluated the diffusion of AAV in mucus from cystic fibrosis patients using the multiple particle tracking analysis.^[^ ^111^ ^]^ The AVV exhibited serotype‐dependent penetration, and the research found that AAV type 6 could cross the mucus rapidly and achieve objective gene transfection in the COPD mice model. Moreover, AAV type 6 has also been used to deliver miRNA‐21‐5p to prevent the type II alveolar epithelial cells (AT2) apoptosis in hyperoxic acute lung injury rats’ model.^[^ ^112^ ^]^ The high efficiency of miRNA‐21‐5p delivery could improve their levels in AT2 and inhibit the PTEN/AKT pathway‐induced cellular apoptosis. Even though the application of AAV is still faced with doubts about its safety, it has exhibited its potential as an effective vector for local disease gene therapy.

### 3.5 Excipients

To achieve the suitable aerodynamic particle size and stability for DPIs, formulated nucleic acids with nanoscale usually need carriers to improve their aerosolization profile and adjust their release behavior. The particular excipients as the carrier could protect the DPIs formulation via spray drying and freeze‐drying to reduce the formulation collapse and nucleic acid leakage. When single excipients hardly cover all the requirements, more excipient combinations may provide desirable formulation features. Several types of excipients have been investigated for their application in developing inhalable nucleic acid formulations, such as saccharides, nucleic acids, and fumaryl diketopiperazine (FDKP).

#### 3.5.1 Saccharides

Due to their excellent safety and economy, saccharides are the most commonly investigated and applied excipients in DPI development. Among them, lactose, mannitol, and trehalose are several sugars being mostly studied in DPI preparation. The FDA and European Medicines Agency (EMA) have approved the lactose‐ and mannitol‐based DPIs for pulmonary administration of small molecules on local disease treatment. The saccharides majorly work as carriers to improve the dispersion of gene‐loaded formulations, and the key parameters when preparing DPIs via spray drying were the hygroscopicity and glass transition temperature (T_g_).

Lactose is the most commonly applied excipient in marked DPIs with confirmed safety and various grades due to different manufacturing processes. Due to the hygroscopicity of spray‐dried lactose in an anhydrous form, the inhalable lactose particles are generally prepared with particle sizes larger than the inhalable range to maintain their monohydrate state. Moreover, the anhydrous lactose as a carrier significantly reduced the drug deposition efficiency due to their high‐energy surfaces, which have stronger adhesive interactions than the lactose monohydrate. The micrometer‐size powder containing nanoscale gene‐loaded formulation could adhere to the rough surface of large lactose particles and be released in an inhaler after dispersing. Most lactose will deposit in the mouth and throat, and only limited lactose with fine particles reaching the lungs are absorbed in epithelium cells. The pulmonary absorbed lactose combined with the lactose swallowed into the digestive tract could be metabolized by intestinal enzymes or intestinal epithelium, which exhibited good safety (up to 25 mg) even in patients with lactose intolerance. Moreover, the sugar‐associated reducing function may also affect the absorbed formulations and the stability of gene‐loaded formulations. Zimmermann et al. reported using 5% lactose solution spray drying with siRNA‐loaded LNPs formulated with Dlin‐MC3‐DMA, helper lipid, cholesterol, and DMG‐PEG.^[^ ^113^ ^]^ Spray‐dried formulation with less than 5% moisture residue and RNA loss below 15%. The lactose‐based DPI could penetrate the lung mucus layer and maintain the bioactivity of siRNA by downregulating over 90% of target protein expression.

Except for the lactose, other saccharides have also been investigated as potential excipients on nucleic acid pulmonary delivery. Among them, mannitol is the most positive potential carrier instead of lactose. Compared to other sugars such as glucose monohydrate, trehalose, dextrose, maltose, and sorbitol, mannitol exhibited stronger hygroscopic resistance with less capillary force in forming mannitol‐based carriers. The hygroscopic sugar could attract moisture, subsequently leading to cohesion and aggregation, significantly reducing effective pulmonary deposition. Keil et al. investigated the effect of mannitol and trehalose on the characteristics of spray‐dried particles that encapsulated siRNA‐loaded polyplexes.^[^ ^114^ ^]^ The investigation found that spray drying with mannitol exhibited lower siRNA recovery but higher FPFs than spraying with trehalose. The spray drying of polyplexes with mannitol could successfully transfect primary T cells and achieve GATA3 silencing. Moreover, it's been proved that inhaled mannitol could stimulate mucociliary generation and mucus clearance, which could be another factor affecting the absorption of pulmonary deposited drugs.^[^ ^115^ ^]^ Kumari et al. reported mannitol conjugation could disrupt the mucin interaction and decrease the viscosity of mucus by increasing the aqueous penetration, which improved the nucleic acid transfection in vitro.^[^ ^116^ ^]^

Trehalose is another sugar that could be used not only as a carrier but also applied in nucleic acid formulation freeze‐drying process, which is based on its high T_g_, the temperature at which glassy materials transform to a rubbery state. With a temperature higher than T_g_, the formulation powder is transformed from a stable glass state to an unstable rubber state with the system viscosity rapidly decreasing, followed by shrinkage, microscopic flow, or even collapse in the porous layer. With high Tg and low hygroscopicity characteristics, trehalose is considered the potential excipient to act as a DPI carrier or lyoprotectant in the nucleic acid formulation freeze‐drying process. Agnoletti et al. reported a DPI formulated of siRNA‐loaded dendrimer nanocomplexes spray‐dried with different saccharide excipients.^[^ ^117^ ^]^ They found siRNA integrity and bioactivity were retained after the spray drying process, and stabilization of nanocomplexes was improved by co‐spraying with trehalose and inulin as excipients compared to spray with crystalline mannitol. Inulin‐incorporated trehalose improved the T_g_ and exhibited a strong water replacement and kosmotropic effect, providing an economic preparation assay for the engineering of dry powder formulations suitable for pulmonary siRNA delivery. Miao et al. also reported using LNPs to prepare soft mist inhaler (SMI) for encapsulated mRNA pulmonary delivery.^[^ ^118^ ^]^ The investigation found that a trehalose‐incorporated buffer system could improve the entrapment efficiency and particle size of LNPs, providing a successful example of their application in aqueous formulation pulmonary delivery of nucleic acid.

#### 3.5.2 Amino Acids

Amino acids have been considered alternative excipients with the function of improving nucleic acid stability or formulation flowability. As an essential nutrient for the human body, amino acids exhibited no safety concerns as excipients in forming DPI formulations. Due to their hydrophobicity, several amino acids have been confirmed to reduce the hygroscopicity of DPI powder, such as leucine and isoleucine. Spray drying leucine with nucleic acid formulation to prepare inhaled dry powder could significantly improve their aerosol performance and in vitro deposition profile. Due to the surfactant‐like behavior of leucine, spray‐dried particles exhibited less aggregation, which partly reduced the powder size during atomization. Xu et al. reported a DPI formulation by spray drying TNF‐α siRNA‐loaded LNPs with leucine, trehalose, and dextran as powder excipients.^[^ ^119^ ^]^ The incorporated leucine accumulated on the surface of powder particles and provided a wrinkled surface morphology to reduce the particle interaction, improving the FPF and aerosol properties. Similar results were confirmed by Mohamed et al.^[^ ^120^ ^]^ They spray dry L‐leucine and mannitol as DPI excipients to encapsulate the miRNA‐146a‐loaded polymeric nanoparticles. The data suggested excipients with an L‐leucine and mannitol ratio of 1:3 exhibited an optimized spray‐drying yield, particle size, and low moisture content. The DPIs with L‐leucine incorporated showed higher FPF and biological activity of miRNA‐146a in vitro, suggesting the advantages of leucine in DPI application during nucleic acid pulmonary delivery.

The effect of amino acids on nucleic acid was also investigated. Amino acids with the extra carboxyl and amino groups in the side chain may interrupt the interaction of positive carriers and nucleic acid, reducing the formulation stability and bioactivity of the gene. Li et al. investigated the effect of different amino acids on aerosolizing gene vectors containing DPI.^[^ ^121^ ^]^ They found aspartic acid and threonine in the DPI formulation reduced the in vitro transfection efficiency of the lipid/polycation/pDNA system due to the competition gene with the vector at the neutral pH environment. The isoelectric points of aspartic acid, threonine, and phenylalanine were 2.77, 5.64, and 5.48, respectively, which carry negative charges and disrupt the gene‐vector complex, followed by reducing the pDNA encapsulation. In contrast, the amino acid carries positive charges during preparation and does not affect formulation structural integrity. Arginine‐incorporated DPI powders maintain the in vitro transfection efficiency after spray drying and improve the aerosolization behaviors with higher FPF.

#### 3.5.3 Fumaryl Diketopiperazine

Besides the most commonly applied saccharides and amino acids, investigators developed other inhalable pharmaceutical excipients as DPI carriers for drug inhalation delivery. Technosphere technology is a novel, multifunctional pulmonary delivery system using a fumaryl diketopiperazine (FDKP) as a carrier.^[^ ^122^ ^]^ FDKP is a pharmaceutical excipient that the FDA has approved to create the commercial product Afrezza for insulin pulmonary delivery. With the acid side chain substituent, FDKP solution exhibited a pH‐responsive dissolution, which has high solubility at pH not lower than 6 while forming an array of microcrystalline plates followed by self‐assemble into microparticles under acidic conditions. Based on the specific characters, our research groups developed inhalable FDKP microparticles and effervescent FDKP microparticles for pulmonary delivery of azithromycin, which suggested improvements in aerosol ability and aerodynamic behavior.^[^ ^123^, ^124^ ^]^ More applications of FDKP based on Technosphere technology were also investigated for biomedicine or small molecules pulmonary delivery against COPD, asthma, and lung infections. Due to the simplified modification process of the side chains, another series of diketopiperazine substitutes will be developed soon, which might be the subsequent advanced progress in the inhalable pharmaceutical excipient investigation.

## 4 Advanced Delivery Strategies to Improve the Pulmonary Bioavailability of the Inhaled Nucleic Acid

The pulmonary bioavailability of inhaled nucleic acid is the critical indicator that affects the therapeutic efficiency of inhaled nucleic acid. By improving the effective pulmonary deposition, reducing the biological clearance, and increasing the cellular transfection, the local transfection efficiency of nucleic acid could effectively be improved for pulmonary administration.

### 4.1 Drug Delivery Systems Development

#### 4.1.1 Improve Effective Pulmonary Deposition

**Figure 5:**
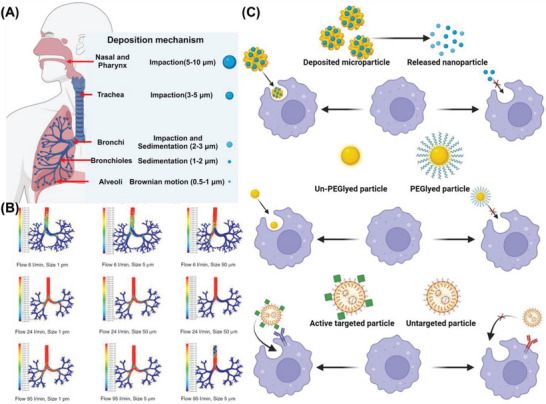
Improve effective pulmonary deposition and reduce macrophage clearance. A) Schematic representation of the respiratory tract and aerodynamic size‐dependent pulmonary deposition. Created with BioRender.com. B) Results of the simulation with computational fluid dynamics (CFD) of particle behavior at different sizes (1 µm, 5 µm, and 50 µm), which are dragged by flows of 6, 24, and 95 L min^−1^. The red areas indicate a high density of trapped particles. Reproduced with permission.^[^ ^125^ ^]^ Copyright 2012, Sociedad Española de Neumología y Cirugía Torácica (SEPAR). C) Strategies avoid alveolar macrophage phagocytosis of deposited particles: particles with size in microscales, unmodified hydrophobic surface, or decorated with active targeting ligands could be effectively swallowed by alveolar macrophage, while the particles with reduced nanoscale size or surface modified by hydrophilic layer could effectively reduce macrophage phagocytosis. Created with BioRender.com. Note: There is a text error in the cited image (Figure B), which we get the copyright to use but have no right to edit, which the Center should be Flow 24 L min^−1^, Size 5 µm, while the Right‐bottom should be Flow 95 L min^−1^, Size 50 µm.

The deposition of inhaled nucleic acid was affected by the characteristics of delivery systems, such as aerodynamic particle size and shape. Based on the previous knowledge, nucleic acid‐loaded formulations should be prepared with aerodynamic size ≈0.5–5 µm to maximize their pulmonary deposition. The primary assay to prepare the genetic drug‐loaded formulations to meet the size requirement was incorporating the inhaled excipients with the genetic drug‐loaded nanoparticles. Spray drying, spray freeze drying, and thin‐film freeze‐drying have been considered effective methods to maintain the genetic cargo integrity during the preparation and control their powder size, shape, and morphology to meet the requirement for effective pulmonary deposition (**Figure** **5**).

Wu et al. reported inhalable siRNA dry powder by spray drying EGFP‐siRNA with mannitol.^[^ ^126^ ^]^ They confirmed that suitable drying parameters, such as inlet temperature and intensive atomization conditions, could affect the particle shape and dispersion, which could reduce their pulmonary deposition. The improved inlet temperature and shear stress also could reduce the integrity of siRNA, while still up to 80% of intact siRNA could be reserved in relatively harsh conditions (180 °C of inlet temperature and 473 L min^−1^ of atomizing gas flow rate), suggesting spray drying a considerable assay for inhalable genetic drug preparation. Chow et al. also introduced a similar inhalable siRNA formulation by spray‐drying naked siRNA with mannitol and L‐leucine.^[^ ^127^ ^]^ The L‐leucine incorporation improved the aerodynamic performance, with the FPF of over 45% and an emitted fraction (EF) of over 80%. There was no observable degradation of siRNA after the spray drying process, suggesting a promising inhaled genetic drug‐loaded dry powder for further investigation.

Except for the naked nucleic acid, genetic drug‐loaded particles could also be applied to prepare inhalable dry powder with excipients. Nucleic acid could be loaded in nanoscale particles first and then prepared microscale particles after spray‐drying with amino acids and saccharides. Mohamed et al. introduced a DOTAP/PGA‐PDL nanoparticle for miRNA‐146a effective loading and integrity preservation.^[^ ^120^ ^]^ Followed by a spray dried with L‐leucine and mannitol at an inlet temperature of 70 °C, the inhalable miRNA‐146a dry powder could effectively maintain its biological activity compared to the freshly prepared miRNA‐146a‐loaded DOTAP/PGA‐PDL nanoparticles. Other similar nano‐in‐micro designs were observed for siRNA and pDNA pulmonary delivery, preparing nanoparticles using lipid materials, polymeric materials, and peptides, followed by spray drying with mannitol, lactose, or trehalose to prepare microparticles,^[^ ^85^, ^128^, ^129^, ^130^ ^]^ exhibited the similar advantages in improving formulation pulmonary deposition with elevated FPF and emitted fraction (EF).

Spray freeze drying (SFD) is also an attractive assay for preparing inhaled genetic drugs by avoiding exposure to elevated temperatures during formulation and improving their stability. Liang et al. reported using a special two‐fluid nozzle to produce naked siRNA SFD powders.^[^ ^131^, ^132^ ^]^ The siRNA powder exhibited a highly porous morphology with an aerodynamic diameter of ≈4.1–4.5 µm. The optimized naked siRNA powder with an EF above 90% and FPF of 30% suggests a significant improvement in genetic drug pulmonary deposition prepared by SFD assay. Thin‐film freeze‐drying (TFFD) is another novel assay for inhalable dry powder preparation. Wang et al. designed an aerosolize siRNA‐loaded solid lipid nanoparticle (SLNs) for potential pulmonary delivery.^[^ ^98^ ^]^ The siRNA/DOTAP complex was mixed with lecithin, cholesterol, and PHC to prepare SLNs, and the mannitol was further mixed with the SLNs suspension, followed by dropping on a rotating, pre‐cooled stainless steel drum to freeze the mixture. After lyophilization, inhalable siRNA dry powder was collected. The TFFD‐prepared dry powders exhibited a porous and fluffy surface structure, thus exhibiting a low MMAD (3.60 ± 0.43 µm) and excellent aerosol performance with FPF of 44.50 ± 5.6% with the unchanged siRNA biological activity. The data indicating TFFD‐prepared siRNA‐loaded SLNs could be a promising method for improving the genetic drug accumulation in the lung.

#### 4.1.2 Overcome Pulmonary Clearance

Macrophage phagocytosis is a significant clearance mechanism for the inhaled genetic drug‐loaded particles after being deposited into the lungs. To improve the cellular uptake in pulmonary epithelial cells, drug carrier was always designed to prolong the pulmonary retention after administration. Therefore, overcoming macrophage recognition and phagocytosis is a vital assay improving the formulation effectively delivery of loaded nucleic acid.

Even though the particle with an aerodynamic diameter of 0.5 to 5 µm exhibited better pulmonary deposition, particles in this size range are also the most easily phagocytosis by the alveolar macrophage. It has been reported that macrophage clearance occurred within 30 min after pulmonary administration of formulations, indicating the low pulmonary delivery efficiency of therapeutic agents, including nucleic acid. To achieve the pulmonary sustained drug release, Yan et al. reported a zinc‐ions coordinated carboxymethyl chitosan‐hyaluronic acid microgel for biological agents (such as BSA) pulmonary delivery.^[^ ^133^ ^]^ With the hyaluronic acid modification, the inhaled microgel with a size of ≈4 µm could avoid the macrophage uptake in vitro and achieved a sustained release up to 36 h after being administrated into mice lungs, which consistent with the previous research, suggesting hyaluronic acid could reduce alveolar macrophage phagocytosis of the deposited formulation.^[^ ^134^ ^]^ The nucleic acid, as a biological therapeutic agent with negative charges, also exhibits the potential to be loaded and effectively delivered into the lung using a similar design.

The particle size was not the only factor that induced alveolar macrophage phagocytosis. The solubility and rigidity of inhaled particles also powerfully affect their fate when incubating with macrophages. Shen et al. reported phagocytosis escaped cholesterol‐conjugated polyoxyethylene sorbitol oleate (CPSO) micelles for pulmonary delivery of small molecule paclitaxel (PTX).^[^ ^135^ ^]^ They declare that polysorbate 80 could significantly improve the solubility of drug‐loaded micelles and reduce their structure rigidity compared to the PLGA nanoparticle, which effectively avoids alveolar macrophage phagocytosis. Adopting a similar design scheme, carriers with hydrophilic chain modification to improve the surface solubility and deformability could be a selective assay against pulmonary macrophage clearance and elevate the delivery efficiency of genetic drugs.

Some specific receptors have been proven to be highly expressed on the macrophage surface, which also could induce the potential ligand‐mediated macrophage phagocytic clearance. Mannose receptor C type 1 (MRC1), also named CD206, has been reported mainly expressed on the immune cells, including macrophages, to recognize the terminal mannose, N‐acetylglucosamine, and fucose residues of bacterial surface polysaccharides and improved their clearance, thus limited the inhaled excipient application such as mannose and glucose. To overcome the obstacle, our research reported a fumaryl diketopiperazine (FDKP)‐based effervescent microparticle for drug pulmonary delivery to avoid macrophage phagocytosis.^[^ ^124^ ^]^ FDKP, as a novel excipient without sugar structures, could prevent MRC1‐based macrophage recognition. The FDKP effervescent microparticle could be disintegrated into smaller fragments during inhalation, significantly improving their pulmonary deposition in the deep lung. Moreover, the highly water‐soluble FDKP could be rapidly dissolved into alveolar mucus and released encapsulated drugs or secondary drug carriers, which also helps avoid alveolar macrophage recognition and reduced therapeutic agent clearance.

As one of the major functions of macrophages was phagocytosis aging or disfunction red blood cells, using the blood cell membrane coating the nanoparticle could be another way of drug‐loaded carrier to avoid being recognized by the macrophages. Lv et al. reported a nanoparticle prepared by compressing PD‐L1 siRNA with lipofectamine 2000. The lipo2000‐siRNA complex further fused with the red blood cell membrane and modified with AS1411 aptamer to provide the non‐small‐cell lung cancer targeting ability.^[^ ^136^ ^]^ The in vitro investigation confirmed red blood cell membrane‐endowed lipo2000‐siRNA complex lower cytotoxicity and escape from macrophage phagocytosis. Due to the excellent ability to avoid clearance, the red blood cell membrane, as a potential biomimetic material, could be further applied in other lipid materials for inhaled delivery cargo development and genetic drug‐effective pulmonary delivery.

#### 4.1.3 Enhance Mucus Penetration

**Figure 6:**
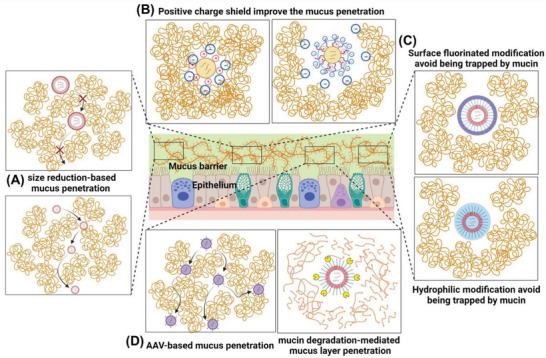
Strategies to overcome the mucosal barrier and improve mucus penetration: A) The deposited particles with size not larger than low‐viscosity channels or pores within the mucus could rapidly penetrate the respiratory mucus layer; B) Particles with neutral or negative surface charges could avoid the mucus traction due to the electrostatic repulsion; C) Particle surface modification with a fluorinated layer or hydrophilic layer (PEGylation) could be isolated from the interaction with the mucus layer; D) Nucleic acid delivery using virus‐based carrier or co‐delivery with mucin degradation agents could achieve mucus penetration effectively. Created with BioRender.com.

Successful deposition of drug‐loaded carriers was not termination of their fate after administration. For transportation in the targeted cells, drug carriers still need to go across the sticky mucus layer to meet the cells. The viscosity of bronchus mucus is derived from the secreted glycoproteins by the epithelial cells for protection from foreign particles and toxins. The particle size, surface charge, and surface hydrophilicity are the potential factors that influence the mucus penetration of particles. To overcome the mucus barrier, several delivery systems have been precisely designed to reduce the mucus bondage, an effective assay improving the transfection of genetic drugs (**Figure** **6**).

The secreted glycoproteins with long chains could interleave with each other and form gelation networks with nanoscale holes for soluble substance transportation. Therefore, researchers proposed that cutoff hole size in the gel network could be the key factor affecting drug‐loaded particle penetration of the mucus layer. Several studies have reported that particles with a size larger than 200 nm hardly cross the mucus layer.^[^ ^137^ ^]^ Ravikumar and co‐workers reported PLGA nanoparticles loading EpoR‐cDNA for pulmonary delivery using a nebulizer with an acceptable cellular uptake both in vitro and in vivo.^[^ ^138^ ^]^ The cDNA‐loaded PLGA‐NPs with particle size 196 ± 36 nm could transport into epithelial cells after pulmonary administration and effectively express EpoR.

To effectively load the nucleic acid in the formulations, the carriers always exhibited positive charges for binding genetic drugs via electrostatic adsorption. However, cationic polymeric or lipid materials could not only effectively encapsulate nucleic acids but also easily interact with glycoproteins in mucus and be trapped inside. Therefore, reducing the surface charges of delivery vectors via designing novel materials or delivery systems could be an option for effectively crossing the mucus layer. PEGylation coating on the surface is a classic assay to reduce the positive charges of particles. Mastorakos and co‐workers designed a novel biodegradable PEGylated poly(β‐amino esters) (PEG‐PBAEs) for DNA condensation.^[^ ^75^ ^]^ Compared to the non‐PEGylated PBAE particles, PEG‐PBAE‐based NPs reduced their surface charges to near‐neutral (decreased from 30 to 2 mV). The PEG‐PBAEs‐NPs effectively penetrated the human mucus gel layer and reached the underlying epithelium. The in vivo transfection study confirmed the PEG‐PBAE‐NPs mediated a significant transgene expression compared to non‐PEGylated PBAE particles and PEI‐NPs. Grun et al. evaluated the effect of PEGylation on the transfection efficiency of mRNA in vivo.^[^ ^139^ ^]^ They designed a PEG‐conjugated poly(amine‐co‐ester) (PEG‐PACE) for the mRNA pulmonary delivery and prepared the formulation with different percentages of PEGylated PACE. The data suggested PEGylated could improve the transfection efficiency of mRNA after pulmonary with a concentration lower than 5% wt., indicating the PEGylation of cationic vehicles must be optimized by PEG content, cargo type, and delivery route. Anyway, the PEGylation on the surface is still a potential design to reduce the overall charges of the delivery carrier. Except for using PEGylation, other surface modification assays may also exhibit similar potentials to reverse surface charges and improve mucus penetration. Fukushige et al. reported an HA‐coated liposome‐protamine‐gene complex (LPDH) for nucleic acid pulmonary delivery.^[^ ^63^ ^]^ Compared to the non‐hyaluronic acid‐modified formulation with charges of 31.7 ± 1.5 mV, LPDH exhibited a reduced surface charge to −13.1 ± 4.5 mV, significantly decreasing their cytotoxicity. Even though there was no mucus penetration data study in this project, we can still expect the effective mucus barrier crossing ability of LPDH, which exhibited an improved cellular uptake and targeted gene silence in vitro. A similar design was applied by Zhu et al., who developed an HA‐dopamine‐coated PBAE nanoparticle for TNF‐α siRNA pulmonary delivery.^[^ ^39^ ^]^ Their data suggests that HA‐dopamine coating forms a charge screen shell to shield the positive charge of PBAE/siRNA, decreasing from 10 mV to almost neutral. The mucus penetration assay confirmed that PBAE particles with HA‐dopamine coating exhibited a higher mucus transportation rate. They also propose the dopamine‐based adhesive property also facilitates nanoparticles across the mucus layer. Kumari et al. reported using the hydrophilic biopolymer chondroitin sulfate A (CS‐A) conjugated with a mucolytic agent, mannitol, to improve the mucus permeability to deliver siRNA or pDNA effectively.^[^ ^116^ ^]^ The hydrophilic CS‐A coating could reduce the surface charges and decrease the interaction with mucins due to electrostatic repulsion. At the same time, the conjugated mannitol could disrupt the hydrogen bonds between mucins and improve their fluidity. Yang et al. reported using RC peptide, an inflammation‐sheddable and charge‐reversal pro‐peptide of RAGE‐binding peptide, to modify polylysine/siRNA nanocomplex. The negative charges of RC peptide reduced the mucin interaction due to the electrostatic repulsion.^[^ ^38^ ^]^ All the above research cases suggested the effects of regulating surface charges in improving particle mucus penetration.

Except for the surface charge, the surface hydrophilicity was also observed to affect the mucus penetration ability of particles strongly. PEGylation of the inhalable genetic drug carriers could also improve their hydrophilicity, followed by enhancing their mucus penetration. Kim et al. compared the transfection efficiency of ENaC‐silencing plasmids loaded with PEGylated PBAEs and non‐PEGylated PBAEs.^[^ ^140^ ^]^ After intratracheal administration of formulation in a cystic fibrosis transgenic mouse model (*Scnn1b‐Tg* mice), PEGylated PBAEs exhibited around 8‐fold greater transgene expression compared to non‐PEGylated PBAEs in the alveolar region. Kim and his co‐worker also observed a similar phenomenon. They optimized the formulation of commercial Onpattro by adjusting the amount of DSPC and DMG‐PEG and types of sterol.^[^ ^14^ ^]^ The data confirmed that DMG‐PEG concentration in LNPs is positively related to their mucus penetration ability and shear resistance, suggesting that PEGylation is an attractive design concept in mucus‐penetrated drug delivery carrier development. Even though many other researchers have also reported similar effects of PEGylation in particle mucus crossing,^[^ ^141^ ^]^ there are also different sounds. Conte et al. provided an opposite conclusion about the impact of PEGylation.^[^ ^142^ ^]^ They compared the pulmonary mucus permeability of an NF‐κB siRNA‐loaded PLGA nanoparticle coating with DPPC or DSPE‐PEG. The data found that PEGylation has not boosted cystic fibrosis mucus penetration, which may be due to the existence of the biomimetic lipid coating shell. This research provided an alternative choice in designing a mucus‐penetrating nucleic acid delivery system.

Guan and co‐workers have reported respiratory mucus permeable peptide‐poloxamine nanoparticles for mRNA and plasmid DNA transfection both in vitro and in a cystic fibrosis mouse model.^[^ ^143^ ^]^ They designed a multi‐modular peptide and poloxamine 704 for various nucleic acid loading via self‐assembly and evaluated in vivo transportation efficiency. The small size (<100 nm) and high density of hydrophilic brushes due to hydrophilic PEO blocks in particle surface was the potential reason for the effective mucus penetration in vivo. Moreover, they used a similar delivery system in the pulmonary delivery of Spike protein coding pDNA to induce mucosal immunity.^[^ ^144^ ^]^ Their investigation indicated a promising respiratory mucus penetration and nucleic acid pulmonary delivery platform. Phage original peptide modification is also being investigated. Leal et al. identified phage source peptides with hydrophilic and neutral charges, founding peptides CGGQDLKSC and CPSSSREKC modification could effectively improve the particle mucus penetration.^[^ ^145^ ^]^ Yang et al. reported using RC peptide, an inflammation‐sheddable and charge‐reversal pro‐peptide of RAGE‐binding peptide, to modify polylysine/siRNA nanocomplex. The negative charges of the RC peptide reduced the mucin interaction due to the electrostatic repulsion.

The fluorinated modification was discussed a long time ago to improve the gene transfection efficacy of lipid or polymeric vectors.^[^ ^146^, ^147^ ^]^ The fluorinated materials exhibit serum resistance because they have lipophobic and hydrophobic features, which are against the serum protein absorption on their surface. A similar phenomenon is expected to occur in the pulmonary mucus layer via reducing mucus glycoproteins interaction and adsorption.^[^ ^148^ ^]^ Ge et al. designed and synthesized guanidinated and fluorinated bifunctional helical polypeptides to form polyplexes with TNF‐α siRNA. The mucus permeability of polypeptides with various fluorinated chain lengths was all evaluated, and data suggested that polypeptide P7F7 provoked the highest TNF‐α knockdown with its silence rate of ≈96%. This investigation provided an effective transmucosal gene delivery strategy for pulmonary disease treatment.

Except for modifying the carrier to meet the requirement of mucus penetration, some researchers also designed a mucus‐degradable carrier for nucleic acid across the mucus barrier. Charbaji et al. designed a disulfide‐containing linker conjugated dendritic polyglycerol (dPG)‐based nanogel and further participated in poly‐N‐isopropylacrylamide (PNIPAM) and poly‐N‐isopropylmethacrylamide (PNIPMAM) polymerization.^[^ ^149^ ^]^ The synthesized nanogels carrying TNF‐α siRNA exhibited an improved mucosal barrier penetration in vivo compared to the nanogels without disulfide bond linker, suggesting the disulfide bond in nanogels could degradable crosslinked mucus glycoprotein and improve the fluidity of mucus layer, followed by enhancing particle penetration. Sharma used a similar design mentality by preparing an N‐acetylcysteine (NAC) modified PLGA microparticle to improve the pulmonary bacterial biofilms.^[^ ^150^ ^]^ Enhanced NAC‐PLGA particle diffusion in mucus was observed, and the mucus viscosity was reduced after co‐incubation, suggesting their mucus degradation ability with advances in effective pulmonary drug delivery.

Adeno‐associated viral vector has also been found to improve the mucus layer penetration, thus also being investigated as a genetic drug carrier for pulmonary delivery.^[^ ^111^ ^]^ Duncan et al. reported that AAV serotype 6 can rapidly diffuse through mucus from cystic fibrosis patients, which exhibited a capsid mutation to avoid the mucus mesh adhesion and will not induce mucus secretion.^[^ ^111^ ^]^ Ferreira et al. reported a tyrosine‐mutant AAV8 (Y733F‐AAV8) for effective mucus diffusion in a mouse model.^[^ ^151^ ^]^

Except for surface modification, the shapes of the delivery carrier could also affect the mucus penetration behavior. Bao and co‐workers designed five types of amphiphilic α‐lactalbumin (α‐lac) peptides‐based peptosomes with various shapes and rigidities and investigated their mucus penetration in intestinal mucus.^[^ ^152^ ^]^ They found that shapes of short nanotubes exhibited improved permeability in mucus compared to shapes of long nanotubes, spheres, and crosslinked short nanotubes, which might be due to their smaller size and flexible tube structure. Even though this investigation was applied to the intestine, we can reasonably expect a similar phenomenon to be exhibited in the pulmonary mucus layer.

#### 4.1.4 Regulate Intracellular Release

**Figure 7:**
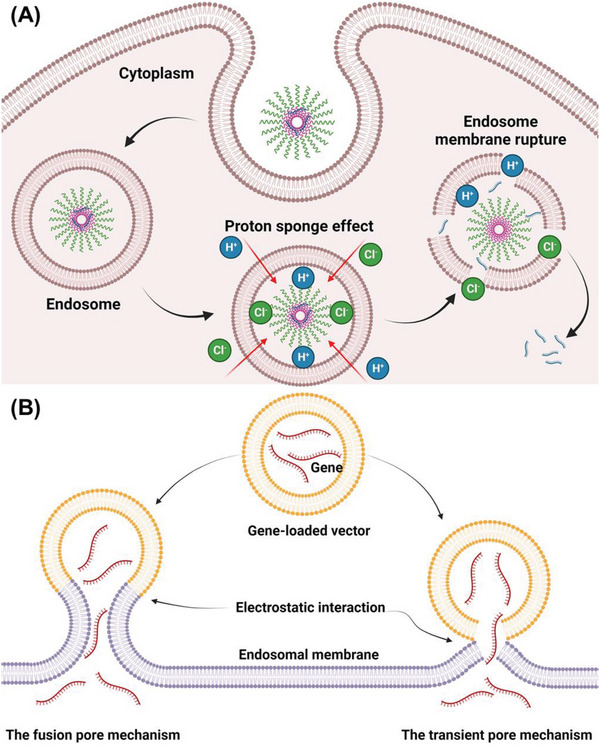
Endosomal escape mechanism of lipid and polymer‐based gene delivery vector. A) Schematic diagram of the proton sponge effect for cationic polymer‐based nanoparticle endosomal escape. B) Schematic diagram of the fusion pore mechanism and transient pore mechanism for cationic lipid‐mediated endosomal escape. Created with BioRender.com.

After overcoming the previous delivery obstacles, the pulmonary drug delivery system is then facing its last difficulty: how to successfully escape from endosome/lysosome and achieve effective intracellular genetic drug release. Even though many efforts have been made to develop inhalable genetic drug delivery systems, there is still a lack of effective methods to improve drug‐loading vectors' endosome/lysosome escape. Therefore, several widely acceptable endosome/lysosome escape mechanisms need to be selectively followed during the novel vector design (**Figure** **7**).

For the vectors composed of polymeric materials, the hypothesis of the proton sponge effect has been considered the possible mechanism for their effective release from the endosome. The cationic polymers with various amine groups exhibited a buffering capacity. After being transferred into the endosome, amine groups bind protons gradually and induce extra protons to be transported into the endosome to maintain the acid environment. At the same time, the chloride ions and water would also enter the endosomes due to the osmotic pressure gradient and charge balance. Based on the above phenomenon, endosomes eventually keep swelling and rupture with the content released into the cytoplasm.^[^ ^153^ ^]^ Ma et al. reported using a star shape polymer (PDMAEMA‐POEGMA) nanoparticles complexed with siRNA for lung cancer therapy.^[^ ^154^ ^]^ The nebulized star‐siRNA nanoparticles with proton sponge effect effectively released into cytoplasm from the endo‐lysosome with an escape rate over 70% at the N/P ratio of 28:1. Miyazaki and co‐workers designed a poly(ethylene glycol)‐poly(glycidylbutylamine) (PEG‐PGBA) polymer for mRNA effective transfection.^[^ ^155^ ^]^ Compared to the classic cationic polymer PEG‐polylysine (PEG‐PLL), PEG‐PGBA exhibited a higher mRNA release into cells and lower gene‐endosome colocalization, suggesting an improved genetic drug endosome escaping performance. Zhu et al. used the HA‐DA‐coated PBAE vectors for siRNA pulmonary delivery. They observed a time‐dependent endosome escape of siRNA‐loaded nanoparticles, which 8 h after incubation with cell, the formulation could be released due to the endo/lysosome rupture by the proton sponge effect.^[^ ^39^ ^]^ Additionally, Gong et al. reported a proton‐driven transformable carrier for biomacromolecules effectively release.^[^ ^156^ ^]^ They designed a series of p(DMAEMA‐OGEMA)‐b‐p((MAVE)‐(MAVE‐NDP)) polymers and optimized their structure to achieve better endosome rupture and transform‐based drug release ability. The polymer could effectively be released from the endosome due to the proton sponge effect, followed by a sphere‐to‐nanofiber or sphere‐to‐nanosheet transform in the cytoplasm, leading to the desorption of the loaded therapeutic biomacromolecules.

Except for the proton sponge effect, there are also other mechanisms for endosome drug release. The lipid‐based materials could achieve endosome escape by two types of hypotheses: the fusion pore model and the transient pore model.^[^ ^157^ ^]^ For the fusion pore model, the cationic lipid from carriers could directly fuse with the endosomal membranes via electrostatic interactions, then releasing contents into the cytoplasm. The transient pore model could be explained by forming the transient pores on the endosomal membranes after interacting with cationic lipids from carriers. The ionizable lipid, as the recent research hotspots, could induce endosome rupture via conformational switching.^[^ ^158^ ^]^ The protonated lipid could interact with the anionic lipids in the endosome membrane, forming an inverted cone shape followed by a hexagonal transformation. Mukherjee et al. reported an LNP system for ENaC mRNA pulmonary delivery. The Dlin‐MC3‐DMA was used as an ionizable lipid to prepare LNPs with cholesterol, DSPC, and DMA‐PEG using a microfluidic assay.^[^ ^159^ ^]^ The LNPs effectively achieve ENaC mRNA intracellular transfection and significantly elevated the ENaC expression, suggesting a potential way for mRNA delivery locally. Jin et al. also designed a novel type of LNP for the pulmonary delivery of siRNA.^[^ ^160^ ^]^ The LNP formation with a novel ionizable lipid 246C10, cholesterol, DSPC, and C16‐PEG‐mannose exhibited efficient delivery of siRNA and down‐regulating the epithelial‐mesenchymal transition (EMT)‐related protein expression. Even though there are various structures of ionizable lipids in different LNP systems, they all share the same endosomal escape mechanism, suggesting an attractive route for mRNA and other genetic drug pulmonary delivery. Additionally, a specific oligo(serine ester)‐based charge‐altering releasable transports (Ser‐CARTs) also has been designed for mRNA effective release.^[^ ^79^ ^]^ The Ser‐CARTs could bind mRNA with oligo(serine ester) and achieve a cytoplasm release due to the oligo(serine ester) rapid rearranging of the neutral serine‐based products via O‐N acyl shifts. Thus, the loaded mRNA could rapidly release from the nanoparticles and improve their transfection efficiency.

### 4.2 Improve the Stability of Nucleic Acid

Improving the stability of inhaled nucleic acid was another key strategy in enhancing their therapeutic efficiency. It's been proved that cationic polymers, peptides, dendrimers, and lipidoid compounds could provide stabilizing effects on nucleic acids such as miRNA, siRNA, mRNA, and RNA adjuvants.^[^ ^161^, ^162^, ^163^ ^]^ Advanced formulation technology has been applied in preparing genetic drug delivery systems, such as designing nanoscale nanoparticles, liposomes, and lipid nanoparticles. Encapsulated nucleic acid could be isolated from the aqueous environment and extracellular DNA or RNA enzymes, which are the major causes of the inactivation of genetic drugs.

#### 4.2.1 Improve Extracellular Stability

A polymeric material‐based polyplex was prepared from the charge interaction between cationic polymer and nucleic acid. With the advantage of easy preparation and surface modification, polyplex has become an important gene delivery vehicle. It's been reported that RNA polyplex stabilization could be improved by increasing the interaction between polymeric materials and RNA. Among them, increasing polymer hydrophobicity by modifying the cholesterol units or alkyl chains in polymer has been proven to improve the stability of RNA‐loaded polyplex during the delivery process.^[^ ^164^, ^165^ ^]^ Polyplexes without hydrophobic units could rapidly accumulate in the kidney and be excreted due to their highly hydrophilic properties. Levačić et al. reported a library of polyplexes of PEI‐like peptides termed sequence‐defined oligoaminoamides (OAAs) for RNA delivery.^[^ ^166^ ^]^ The OAAs modification with fatty acids could improve the stability and lytic activity, further enhancing the RNA transfection efficiency. Kim et al. investigated the effects of hydrophobic moieties in polyaspartamide‐derived polymer side chains on mRNA delivery.^[^ ^167^ ^]^ Data suggested that polymers with high logP values, representing more hydrophobic moieties, could effectively prevent mRNA degradation in the culture medium. Similar data were also reported by other research groups, which used the polymeric‐lipid hybrid nanoparticles content of PBAE, lipid‐PEG, helper lipid, and cholesterol.^[^ ^168^ ^]^ Lipid‐based formulations such as liposomes and LNPs all contain amphiphilic lipid materials, which could effectively encapsulate genetic drugs inside and, therefore, prolong the residence time after administration, exhibiting the advantages of genetic drug delivery. All the data suggested a suitable hydrophobic in vectors could be a strategy for improving the stability of loaded nucleic acid.

The binding affinity between carriers and nucleic acid is also a critical factor that affects formulation stability against RNases. One study compared the mRNA stabilizing effect of guanidine and primary amine in the micelles system.^[^ ^169^ ^]^ Data suggested guanidine could stabilize mRNA‐loaded micelles against the nuclease attack more effectively compared to the primary amine. Another research investigated the nuclease against efficiency using various PEG‐polypeptide/mRNA complexes.^[^ ^170^ ^]^ They found that PEG‐poly(tyrosine) stabilizes mRNA‐loaded micelles from the nuclease, which might be due to the stronger π‐ π stacking between tyrosine and mRNA. The tyrosine‐modified copolymer exhibited more efficient mRNA transfection compared to glycine and leucine‐modified copolymers.

Surface coating of RNA polyplexes with hydrophilic polymers, such as PEG, is another widespread method for protecting polyplexes from RNase attack. A hydrophilic surface could form a thick hydration layer to isolate the RNase's direct interaction with RNAs. However, enough PEG density was the key to their RNase against ability. Yoshinag and co‐workers found that the small RNase size and low PEG density may reduce mRNA stability loaded in the formulation.^[^ ^171^, ^172^ ^]^ They prepared a mRNA‐loaded PEG (12 kDa)‐PAsp(DET) copolymer and evaluated their ability against RNase. After calculation, they found the average distance between adjacent PEG chains was 8.9–9.3 nm, and the radius of gyration of 12 kDa PEG is 4.7 nm, which hardly stops the smaller RNase A (less than 4 nm) from penetrating the core of micelles and degradation of loaded mRNA.

**Figure 8:**
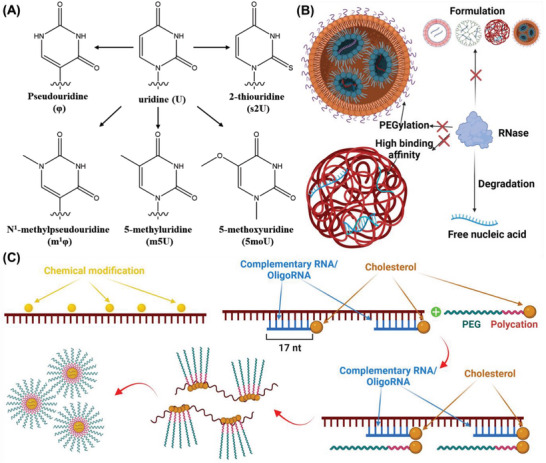
Strategies to improve extracellular stability of therapeutic nucleic acid. A) Chemical modifications of the nucleoside bases to enhance the stability of RNA therapeutics. B) Gene‐encapsulated formulations are designed to improve the stability of RNA therapeutics. C) Improving mRNA stability via chemical modification, complementary RNA/Oligo RNA hybridization, or hydrophobic moieties combination. Created with BioRender.com.

Chemical modification has been considered another strategy that can improve the stability of the nucleic acid on either base groups or backbone. Nucleoside base modification can improve their stability without compromising their binding efficacy, mostly processed by replacing uracil with pseudouridine.^[^ ^173^ ^]^ Karikó et al. reported the incorporation of pseudouridine, Ψ, in mRNAs could confer enhanced translational capacity than the unmodified mRNAs.^[^ ^174^ ^]^ The presence of Ψ in mRNA improved their stability and reduced the immunogenicity in vivo, suggesting a potential mRNA modification for replacing conventional vaccines in force expression of targeted proteins. Other possible uracil modifications include N^1^‐methylpseudouridine (m1Ψ), 5‐methyluridine (m5U), 5‐methoxyuridine (5moU), and 2‐thiouridine (s2U).^[^ ^175^, ^176^ ^]^ Manger et al. reported using 1‐methyl‐pseudouridine (m1Ψ) instead of uridine in mRNA, which induced global changes in RNA's secondary structure and enhanced the expression of the coded protein.^[^ ^177^ ^]^ At the same time, 1‐methyl‐pseudouridine (m1Ψ) replacement also increased base stacking and mRNA's melting point, improving mRNA's stability (**Figure** **8**).

The backbone modification is also being investigated to improve the stability of therapeutic nucleic acid. siRNA could conjugate with stabilization molecules such as cholesterol, which could maintain their knockdown efficiency as long as they suppress burst release and prolong their circulation in vivo.^[^ ^178^ ^]^ Connecting siRNA with polycations via an environment‐responsive linker could also stabilize the gene‐loaded formulations. The pH‐sensitive cis‐maleic acid amide could achieve its endosome target release and exposed polycation to disrupt the endosome membrane and improve siRNA escape.^[^ ^179^ ^]^ Due to their single‐strand structure, mRNA is less stable and hardly achieves effective modification compared to double‐strand nucleic acid. Adding 5′cap and poly‐A tail has been considered the standard assay to improve their stability. Moreover, hybridizing mRNA with stabilizing moieties conjugated complementary RNA oligonucleotides could be another solution. Yoshinaga et al. reported cholesterol (Chol)‐tethered RNA oligonucleotides (Chol (+)‐OligoRNA) pre‐hybridized with mRNA could effectively against the RNase degradation.^[^ ^171^ ^]^ The Chol‐OligoRNA could compress the hybrid mRNA into the hydrophobic core, which reduces the interaction with RNase and effectively improves the targeted protein expression. Additionally, no immune response was observed of the formulation after administration via reducing the length of the OligoRNA reach to 17 nt, suggesting minimizing hybridization length could avoid double‐strand RNA‐mediated immunostimulation.

#### 4.2.2 Improve Intracellular Stability

**Figure 9:**
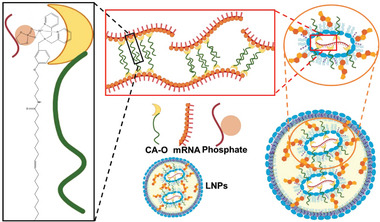
Schematic illustration of CA‐O and ssRNA nano‐formulation to improve their intracellular stability. CA‐O could bind with ssRNA by coordination between the Zn/DPA head and phosphate in the RNA backbone, then encapsulated nucleic acid inside the particles to protect from enzyme‐based degradation. Created with BioRender.com.

The intracellular stability of genetic drug‐loaded formulations was a more complicated issue compared to their extracellular stability. The endosome escaped nucleic acid needs to be released into the cytoplasm to produce their biological effect. Due to the short half‐life of mRNA, it seems prolonging their retention in cytoplasm could be a strategy for improving their transfection efficiency. It's been proved that improving affinity between mRNA and carriers could slow down their release from the formulation, followed by prolonging their function and target protein expression. Park et al. reported a coordinative amphiphile (CA) as the stabilizer of single‐strand RNA.^[^ ^180^ ^]^ The CA with a phosphate‐directing group, zinc‐dipicolylamine (Zn/DPA), could act as a cationic lipid mimic, which binding phosphate groups in the backbone of nucleic acid with positively charged Zn/DPA groups and forming nanoparticles with size around 100 nm. The formulation achieved a sustained release of single‐strand RNA (TLR7/8 activator), effectively activating the APCs and improving the humoral immune response (**Figure** **9**). Dirisala et al. introduced disulfide crosslinking in mRNA polyplex micelles to improve intracellular nuclease tolerability.^[^ ^181^ ^]^ They established PEG‐PLys block copolymers with extra thiolated in side chains by 3‐mercaptopropionyl (MP) groups or 1‐amidine‐3‐mercaptopropyl (AMP) groups. The PEG‐PLys(AMP), with a tighter mRNA packaging in the core, could significantly elevate the mRNA amount intracellular compared to the PEG‐PLys(MP), suggesting cationic charge preservation enhanced the affinity with mRNA and improved the mRNA nuclease tolerability intracellular. Uchida et al. reported a helix foldamer oligopeptide as an mRNA delivery carrier, which could improve their intracellular stability.^[^ ^182^ ^]^ They synthesized arginine oligopeptide and (Arg‐Arg‐α‐aminoisobutyric acid (Aib))_5_ for mRNA delivery. The Aib incorporated peptide could form the helical structures, significantly improving mRNA intracellular stability over 10‐fold higher than OligoArg, indicating the OligoArg‐Aib aims to prolong the protection conferred on mRNA inside cells.

While other researchers exhibited opposite conclusions. Some researchers believe overstabilization of formulation intracellular negatively impacts the biological efficiency of therapeutic nucleic acid via inhibiting RNA release. Environment‐responsive linkers such as disulfide bonds and phenylboronate ester could regulate nucleic acid release. Manickam et al. compared the efficiency of disulfide bonds contained or non‐contained poly(amido amines) (PAA) in mRNA delivery and transfection.^[^ ^183^ ^]^ They found reducible PAA exceeded non‐reducible PAA on mRNA‐coded targeted protein expression efficiency, which is far more significant in GSH high expressed cell lines, indicating that the reductive environment‐responsive cleavage of carriers improved the mRNA translation in the cytoplasm. Inducing carrier rapid degradation could be another strategy for improving the therapeutic efficiency of nucleic acid. Naito et al. introduced a N‐substituted polyaspartamide (PAsp) for mRNA efficacy delivery.^[^ ^184^ ^]^ By adjusting the amine protonation and alkyl spacer length in side chains with 2‐aminoethyl, 3‐aminopropyl, or 4‐aminobutyl moieties, the degradable PAsp with 2‐aminoethyl modification elicited the highest mRNA transfection efficiency compared to others. These investigations suggest the importance of mRNA efficient release in improving their intracellular transfection.

#### 4.2.3 Improve the Storage Stability

The storage stability of mRNA and siRNA were different even though both drugs could be encapsulated in similar lipid formulations. The current mRNA vaccines from both Moderna and Prizer/BioNTech have reported storage temperatures of −80 to −60 °C, while patisiran (Onpattro) as a siRNA formulation allows 3‐year storage at 2–8 °C. To achieve the economical storage conditions, researchers applied various strategies to improve the storage ability of genetic drugs.

Adding excipients has proved to stabilize the mRNA vaccine by Moderna and Pfizer/BioNTech. Excipients in formulation work as osmolytes, pH buffers, or cryoprotectants to adjust the most suitable conditions for RNA stabilization. It has been reported Moderna uses a Tris‐HCl buffer to stabilize nucleic acid via maintaining a pH environment and eliminating hydroxyl radicals.^[^ ^185^ ^]^ Moreover, the selection of buffer systems should also consider the temperature‐dependent pH changes, which have been observed in the sodium phosphate buffer system with a 3.5 pH‐units drop during the freezing process. Histidine buffer exhibited a pH stabilization ability during freezing compared to other buffer systems, only exhibiting a 0.5 pH‐units drop when the formulation solution decreased from 0 to −30 °C.^[^ ^186^ ^]^ Moreover, the pH condition could also affect the hydrolysis of RNA and lipid materials. Even though the preparation of formulations is in an acid environment, the formulation needs to adjust to a nearly neutral pH value to reduce the pain during administration. The pH of the Moderna and Pfizer/BioNTech mRNA vaccines is adjusted between 7 to 8. While mRNA exhibited the most stable pH ≈4 to 5, which leads to the shorter storage time of the vaccine after dissolving in an aqueous solution.

Due to the aqueous environment easily inducing hydrolysis, one of the major mechanisms of RNA degradation, lyophilization of genetic drug‐loaded formulation, could further improve their storage stability. The freeze‐drying of naked mRNA has been investigated and confirmed their degradation prevention in RNA storage. Jones et al. found that mRNA freeze‐drying with trehalose could maintain stability at 4 °C for 10 months.^[^ ^187^ ^]^ While the naked genetic drugs are still inconvenient for direct administration to the patient, frozen dried formulations of encapsulated nucleic acid should be more valuable for further investigation. The freeze‐drying process exposes nanoparticles to stress for the mRNA‐loaded formulations and easily destroys the prepared formulation structure. The application of saccharides as lyoprotectant has been described previously in Section 3.5.1. Shirane et al. reported an alcohol dilution‐lyophilization method to prepare siRNA‐loaded LNPs.^[^ ^188^ ^]^ Sucrose and DMG‐PEG_5k_ were proved to be the optimized choice to maintain the biological activity of loaded siRNA from the freeze‐drying process. Subsequently, they further applied the method in mRNA‐loaded LNP preparation.^[^ ^189^ ^]^

The optimized formulation exhibited a prolonged storage time for at least 4 months at 4 °C, indicating the effects of sucrose as an effective lyoprotectant. Similar data were observed by Muramatsu and co‐workers.^[^ ^190^ ^]^ They introduced efficient lyophilization procedures to prolong the storage of mRNA‐loaded LNPs. Data suggested mRNA‐LNPs do not lose potency after 12 weeks of storage at room temperature or for at least 24 weeks at 4 °C as a lyophilized product with 10% (*w/v*) of sucrose and 10% (*w/v*) of maltose as lyoprotectant. Kim et al. reported mRNA‐loaded LNPs with TT3, Dlin‐MC3‐DMA, DOPE, Cholesterol, and DMG‐PEG_2k_ as components could maintain stability for at least 30 days when storage in RNAse‐free PBS buffer containing 10% (*w/v*) of sucrose at −20 °C.^[^ ^191^ ^]^ However, after lyophilization, the optimized formulation could hardly achieve in vivo transfection efficiency fully equivalent to fresh prepared LNPs, in which the hydrolytic degradation occurs possibly during the resuspension process. Even though, we can still expect that lyophilized LNPs can be safely stored at higher temperatures for months without losing their transfection properties of loaded genetic drugs.

## 5 Nucleic Acid Pulmonary Delivery in Clinical Trials

Several gene delivery systems via pulmonary have been evaluated in clinical trials for respiratory disorders. ALN‐RSV01 was the first siRNA candidate to be administered via the pulmonary route in clinical trials in 2008. Since then, other projects on inhaled RNA therapy have been initiated while still not approved for clinical use.

ALN‐RSV01 is a naked siRNA designed to fight against viral respiratory infection. It was the first inhaled siRNA evaluated in clinical trials with Phase I started in 2007 (NCT00496821), which confirmed the acceptable tolerance of ALN‐RSC01 via intranasal administration. The following Phase IIb clinical trial (NTC01065935) exhibited the effective reduction in RSV infection‐induced bronchiolitis obliterans syndrome in lung transplant patients, an important milestone for inhaled gene delivery. QR‐010 is another single‐stranded RNA antisense oligonucleotide designed for targeting CF conductance regulator (CFTR) by inhalation RNA‐contained isoosmolar solution to improve the lung function of F508del CF patients. A series of clinical trials (NCT02564354, NCT02532764) was investigated and completed in 2017, which has proved their ability to improve respiratory perameters in CF patients.

MRT5005 was an in vitro‐transcribed and unmodified CFTR mRNA encapsulated in LNPs for CF patients. The hyperbranched PBAEs as the delivery carrier could effectively loaded and transfected in the lung after the pulmonary administration with limited off‐target effects. The tested Phase II clinical trial (NCT03375047) confirmed the MRT5005 with acceptable tolerance and improved respiratory parameters (ppFEV1) in CF patients after single‐dose administration, while did not demonstrate a pattern of increased in ppFEV1 after multiple‐dose portion. Even though the FDA granted Fast Track and Rare Pediatric Disease designations for MRT5005 for CF treatment, Translate Bio Inc. has announced the inhaled MRT5005 showed no significant improvement in lung function among the participants in the second midterm analysis. The result suggested the potential formulation components and their pharmacokinetics need further investigation to improve their clinical investigation in the future.

With the mRNA vaccine successfully developed, Moderna Inc. is also working on the inhaled mRNA formulations in SARS‐CoV‐2 and RSV immunization. The mRNA‐1345 has been approved for the Phase III clinical trials by the FDA for targeting RSV using the same LNP formulation of mRNA‐1273 SARS‐CoV‐2 vaccine, containing SM‐102, DSPC, chlestrol, and PEG_2k_‐DMG. The Phase II data has confirmed their safety in patients over 60 years old with a selective inhalation dose. Moreover, Moderna Inc. also reported an mRNA‐LNP vaccine against SARS‐CoV‐2 via nasal vaccination with a similar lipid formulation perclinic. Although the data suggested that high‐dose nasal administration could include similar immune effects as via intramuscular injection, there are still challenges, such as delivery efficiency, dosage, and safety, that need to be overcome before applying for clinical investigation.

Arcturus Therapeutics Inc. also announced that the FDA granted Orphan Drug Qualification to the candidate product ARCT‐032 for the CF treatment. The ARCT‐032 uses the LUNAR technology, which is composed of proprietary “ATX”‐lipids, phospholipids, cholesterol, and polyethylene glycol lipids with the advantages of accelerating metabolism and reducing toxicity in vivo, to encapsulate CFTR‐coding mRNA for its pulmonary delivery for improving CFTR expression and functional recovery of epithelial cells. The Phase I clinical trial is recruiting patients (NCT05712538), and we expect the data to be reported recently.

Although RNA therapeutics exhibited great potential in the respiratory system, we can expect the mature formulations to be approved for clinical application in the near future. At the same time, some crucial questions remain to be addressed before the successful nucleic acid inhalation delivery system is realized. The exact cellular uptake mechanism and their governer factors should be explored. Moreover, the delivery barrier needs to be overcome, such as the mucus barrier and macrophage scavenging system. The technology to protect the integrity of nucleic acid against shear and thermal stresses during the aerosolization and drying process needs to be developed. In summary, developing a safe and efficient delivery system is still the key to successful clinical gene translation.

## 6 Conclusions and Perspectives

In the past few years, enormous gene therapy strategies have received favorable attention and are considered promising ways to treat or cure diseases via genetic regulation. The outbreak of SARS‐CoV‐2 spawned the development of genetic drugs such as mRNA vaccines and their delivery carrier LNPs. Moreover, encouraging results have established the possibility of using biomacromolecules to treat lung diseases, including cystic fibrosis (CF), asthma, COPD, and lung cancer, via pulmonary delivery, which improves the accumulation of therapeutic agents, reduces the dose, and reduces side effects. Therefore, effective pulmonary delivery of nucleic acid as a valuable research direction drove the attention and enormous attempts from pharmaceutic researchers.

Though viral vectors are widely applied in genome‐editing systems delivery in different contexts, they still face the risks of immunogenicity and tumorigenic, limiting their applications. Based on the advances in nanotechnology, non‐viral vectors have been considered promising resolve for nucleic acid effectively pulmonary delivery. Polymeric‐based materials and lipid‐based formulations have been investigated in pre‐clinical research to improve the transfection efficiency of genetic drugs effectively. However, limited drug delivery systems have still been approved for clinic application. The polymeric‐based vectors still face issues with the immunogenicity of itself or degradation products. Meanwhile, they also face the transfection issue together with the lipid‐based vectors, with the bottleneck of low efficiency of endosome escape. Surface modification could improve the delivery behavior of vectors, such as active targeting and cellular uptake. However, it still has not been examined in clinics and is hardly applicable in industrial production. The LNPs for mRNA vaccine transfection also face the problem of low transfection efficiency, suggesting novel delivery systems are urgently being designed. The application of the delivery system for nucleic acid in clinicals needs to be constructed with simple and safe materials, followed by an enhanced effective intracellular release. To achieve breakthroughs, developing novel materials and outstanding structure design will still be the key to solving the obstacles.

The pulmonary drug delivery system has received enormous attention from researchers since the SARS‐CoV‐2 outbreak. Due to the specific physiological features and microenvironment, gene vectors need to balance their characteristics to deliver successfully. For effective deposition in the respiratory region, gene vectors‐encapsulated inhalable particles need their aerodynamic diameter between 1 and 5 µm. At the same time, the deposited particle with a size larger than 200 nm could be easily phagocytotic by alveolar macrophages. Therefore, designing a size variable gene delivery system could be a resolution to meet the conflicting requirements simultaneously. Moreover, the vectors should be able to penetrate the mucus layer covering the respiratory system. Surface modification via forming the surface hydrophilic layer, neutralizing the charges, or destroying mucus integrity could be a selective choice for crossing the sticky mucus. While except the existent obstacles, the pulmonary pathological status may also strongly affect the effective delivery of genetic drugs, such as in CF or COPD patients, with thicker and more sticky mucus layers compared to healthy people. The surface modification of vectors for improving mucus penetration may solve this barrier, and the co‐delivery of mucus degradation agents with disulfide bonds could also be a choice for enhancing vector delivery.

Improving the loaded gene stability and vector integrity during storage conditions could be another aspect of achieving effective nucleic acid pulmonary delivery. The aqueous environment‐induced hydrolysis could be a major factor in the nucleic acid degradation. To simplify the strict storage conditions, the inhaled formulation could be dried to minimize the water content and improve the storage temperature. The formulation needs to be optimized to maintain its integrity during drying and re‐dissolution. Gene modification can also be another choice to improve their stability during the formulation and delivery process. The future development of inhaled gene‐loaded formulations needs comprehensive consideration of the above designing mentalities to achieve effective pulmonary genetic drugs. Collectively, despite many existing challenges, we are optimistic the pulmonary delivery of genetic drugs will provide precise therapeutic strategies for treating pulmonary in the future.

## Conflict of Interest

The authors declare no conflict of interest.

## References

[1] R. Y. K. Chang , M. Y. T. Chow , D. Khanal , D. Chen , H. Chan , Adv. Drug Delivery Rev. 2021, 172, 64. 10.1016/j.addr.2021.02.017 33705876

[2] Z. Deng , W. Gao , F. Kohram , E. Li , T. V. Kalin , D. Shi , V. V. Kalinichenko , Bioact. Mater. 2023, 31, 1. 37593494 10.1016/j.bioactmat.2023.07.022 PMC10432146

[3] J. C. Kaczmarek , A. K. Patel , L. H. Rhym , U. C. Palmiero , B. Bhat , M. W. Heartlein , F. DeRosa , D. G. Anderson , Biomaterials 2021, 275, 120966. 34147715 10.1016/j.biomaterials.2021.120966

[4] M. Qiu , Y. Tang , J. Chen , R. Muriph , Z. Ye , C. Huang , J. Evans , E. P. Henske , Q. Xu , Proc. Natl. Acad. Sci. USA 2022, 119, e2116271119. 35173043 10.1073/pnas.2116271119 PMC8872770

[5] S. Abed , R. Turner , N. Serniuck , V. Tat , S. Naiel , A. Hayat , O. Mekhael , M. Vierhout , K. Ask , A. F. Rullo , Biochem. Pharmacol. 2021, 190, 114577. 33887259 10.1016/j.bcp.2021.114577

[6] R. Li , Y. Jia , X. Kong , Y. Nie , Y. Deng , Y. Liu , J. Control. Release. 2022, 348, 95. 35636615 10.1016/j.jconrel.2022.05.039

[7] C. Wang , J. Zhou , J. Wang , S. Li , A. Fukunaga , J. Yodoi , H. Tian , Signal. Transduct Target Ther. 2020, 5, 248. 33110061 10.1038/s41392-020-00345-x PMC7588592

[8] A. Papi , B. E. Chipps , R. Beasley , R. A. J. Panettieri , E. Israel , M. Cooper , L. Dunsire , A. Jeynes‐Ellis , E. Johnsson , R. Rees , C. Cappelletti , F. C. Albers , N. Engl. J. Med. 2022, 386, 2071. 35569035 10.1056/NEJMoa2203163

[9] R. Zhang , W. Jing , C. Chen , S. Zhang , M. Abdalla , P. Sun , G. Wang , W. You , Z. Yang , J. Zhang , C. Tang , W. Du , Y. Liu , X. Li , J. Liu , X. You , H. Hu , L. Cai , F. Xu , B. Dong , M. Liu , B. Qiang , Y. Sun , G. Yu , J. Wu , K. Zhao , X. Jiang , Adv. Mater. 2022, 34, 2107506. 10.1002/adma.202107506 35146813

[10] Q. Wang , Y. Shen , G. Mi , D. He , Y. Zhang , Y. Xiong , T. J. Webster , J. Tu , Biomaterials 2020, 228, 119575. 31677394 10.1016/j.biomaterials.2019.119575

[11] X. Lin , R. K. Kankala , N. Tang , P. Xu , L. Hao , D. Yang , S. Wang , Y. S. Zhang , A. Chen , Adv. Healthcare Mater. 2019, 8, e1800910. 10.1002/adhm.201800910 30284409

[12] M. Y. T. Chow , Y. Qiu , J. K. W. Lam , Trends Pharmacol. Sci. 2020, 41, 715. 32893004 10.1016/j.tips.2020.08.002 PMC7471058

[13] R. Y. K. Chang , H. Chan , Nat. Biomed. Eng. 2021, 5, 949. 34616049 10.1038/s41551-021-00794-x

[14] J. Kim , A. Jozic , Y. Lin , Y. Eygeris , E. Bloom , X. Tan , C. Acosta , K. D. MacDonald , K. D. Welsher , G. Sahay , ACS Nano 2022, 16, 14792. 36038136 10.1021/acsnano.2c05647 PMC9939008

[15] X. Zhang , K. Xie , H. Zhou , Y. Wu , C. Li , Y. Liu , Z. Liu , Q. Xu , S. Liu , D. Xiao , Y. Tao , Mol. Cancer. 2020, 19, 47. 32122355 10.1186/s12943-020-01171-z PMC7050132

[16] D. Lu , T. Thum , Nat. Rev. Cardiol. 2019, 16, 661. 31186539 10.1038/s41569-019-0218-x

[17] A. Magnani , M. Semeraro , F. Adam , C. Booth , L. Dupre , E. C. Morris , A. Gabrion , C. Roudaut , D. Borgel , A. Toubert , E. Clave , C. Abdo , G. Gorochov , R. Petermann , M. Guiot , M. Miyara , D. Moshous , E. Magrin , A. Denis , F. Suarez , C. Lagresle , A. M. Roche , J. Everett , A. Trinquand , M. Guisset , J. X. Bayford , S. Hacein‐Bey‐Abina , A. Kauskot , R. Elfeky , et al., Nat. Med. 2022, 28, 71. 35075289 10.1038/s41591-021-01641-x PMC8799465

[18] B. Kim , J. Park , M. J. Sailor , Adv. Mater. 2019, 31, 1903637. 10.1002/adma.201903637 PMC6891135 31566258

[19] Z. Liu , S. Wang , C. Tapeinos , G. Torrieri , V. Kankanen , N. El‐Sayed , A. Python , J. T. Hirvonen , H. A. Santos , Adv. Drug Delivery Rev. 2021, 174, 576. 10.1016/j.addr.2021.05.018 34019958

[20] L. K. McKenzie , R. El‐Khoury , J. D. Thorpe , M. J. Damha , M. Hollenstein , Chem. Soc. Rev. 2021, 50, 5126. 33644787 10.1039/d0cs01430c

[21] A. K. Patel , J. C. Kaczmarek , S. Bose , K. J. Kauffman , F. Mir , M. W. Heartlein , F. DeRosa , R. Langer , D. G. Anderson , Adv. Mater. 2019, 31, 1805116. 10.1002/adma.201805116 PMC7490222 30609147

[22] T. Secher , E. Bodier‐Montagutelli , A. Guillon , N. Heuze‐Vourc`h , Adv. Drug Delivery Rev. 2020, 167, 148. 10.1016/j.addr.2020.06.029 32645479

[23] M. Y. T. Chow , R. Y. K. Chang , H. Chan , Adv. Drug Delivery Rev. 2021, 168, 217. 10.1016/j.addr.2020.06.001 PMC7274121 32512029

[24] F. Huang , Q. Zhu , X. Zhou , D. Gou , J. Yu , R. Li , Z. Tong , R. Yang , Adv. Drug Delivery Rev. 2021, 170, 369. 10.1016/j.addr.2020.09.007 32971228

[25] K. Ahookhosh , O. Pourmehran , H. Aminfar , M. Mohammadpourfard , M. M. Sarafraz , H. Hamishehkar , Eur. J. Pharm. Sci. 2020, 145, 105233. 31978589 10.1016/j.ejps.2020.105233

[26] J. Xi , M. Talaat , X. A. Si , P. Han , H. Dong , S. Zheng , Comput. Biol. Med. 2020, 121, 103791. 32568674 10.1016/j.compbiomed.2020.103791

[27] Y. He , Y. Liang , R. Han , W. Lu , J. C. W. Mak , Y. Zheng , J. Control. Release. 2019, 314, 48. 31644935 10.1016/j.jconrel.2019.10.035

[28] N. Osman , K. Kaneko , V. Carini , I. Saleem , Expert Opin. Drug Deliv. 2018, 15, 821. 30021074 10.1080/17425247.2018.1502267 PMC6110405

[29] C. S. Kim , J. Aerosol Med. Pulm. Drug Deliv. 2021, 34, 147. 34152197 10.1089/jamp.2021.29036.csk

[30] C. B. Morrison , K. M. Shaffer , K. C. Araba , M. R. Markovetz , J. A. Wykoff , N. L. Quinney , S. Hao , M. F. Delion , A. L. Flen , L. C. Morton , J. Liao , D. B. Hill , M. L. Drumm , W. K. O'Neal , M. Kesimer , M. Gentzsch , C. Ehre , Eur. Respir. J. 2022, 59, 2100185. 34172469 10.1183/13993003.00185-2021 PMC8859811

[31] Q. Liu , J. Guan , L. Qin , X. Zhang , S. Mao , Drug Discov. Today. 2020, 25, 150. 31600580 10.1016/j.drudis.2019.09.023

[32] R. Hamed , D. M. Schenck , J. Fiegel , Soft Matter 2020, 16, 7823. 32756700 10.1039/d0sm01232g

[33] M. C. Rose , J. A. Voynow , Physiol. Rev. 2006, 86, 245. 16371599 10.1152/physrev.00010.2005

[34] B. Hu , K. O. Boakye‐Yiadom , W. Yu , Z. Yuan , W. Ho , X. Xu , X. Zhang , Adv. Healthcare Mater. 2020, 9, e2000336. 10.1002/adhm.202000336 32597562

[35] P. Prasher , M. Sharma , S. K. Singh , M. Gulati , N. K. Jha , P. K. Gupta , G. Gupta , D. K. Chellappan , F. Zacconi , T. de Jesus Andreoli Pinto , Y. Chan , G. Liu , K. R. Paudel , P. M. Hansbro , B. G. George Oliver , K. Dua , Chem. Biol. Interact. 2022, 365, 110048. 35932910 10.1016/j.cbi.2022.110048

[36] E. Meziu , M. Koch , J. Fleddermann , K. Schwarzkopf , M. Schneider , A. Kraegeloh , Int. J. Pharm. 2021, 597, 120238. 33540010 10.1016/j.ijpharm.2021.120238

[37] E. Meziu , K. Shehu , M. Koch , M. Schneider , A. Kraegeloh , Int. J. Pharm. X. 2023, 6, 100212. 37771516 10.1016/j.ijpx.2023.100212 PMC10522980

[38] J. Yang , S. Duan , H. Ye , C. Ge , C. Piao , Y. Chen , M. Lee , L. Yin , Adv. Funct. Mater. 2021, 31, 2008960.

[39] J. Zhu , M. Guo , Y. Cui , Y. Meng , J. Ding , W. Zeng , W. Zhou , ACS Appl. Mater. Interfaces. 2022, 14, 5090. 35060376 10.1021/acsami.1c23069

[40] Y. Guo , Y. Ma , X. Chen , M. Li , X. Ma , G. Cheng , C. Xue , Y. Y. Zuo , B. Sun , ACS Nano 2023, 17, 2813. 36719858 10.1021/acsnano.2c11147

[41] J. Watchorn , A. J. Clasky , G. Prakash , I. A. E. Johnston , P. Z. Chen , F. X. Gu , ACS Biomater. Sci. Eng. 2022, 8, 1396. 35294187 10.1021/acsbiomaterials.2c00047

[42] Adivitiya, M. S. K , S. Chakraborty , S. Veleri , S. Kateriya , Biology 2021, 10, 95. 33572760 10.3390/biology10020095 PMC7911113

[43] Y. Cao , M. Chen , D. Dong , S. Xie , M. Liu , Thorac. Cancer. 2020, 11, 505. 31975505 10.1111/1759-7714.13323 PMC7049516

[44] J. Li , H. Zheng , X. Li , J. Su , L. Qin , Y. Sun , C. Guo , M. Beck‐Broichsitter , M. Moehwald , L. Chen , Y. Zhang , S. Mao , Eur. J. Pharm. Biopharm. 2019, 143, 70. 31446045 10.1016/j.ejpb.2019.08.017

[45] E. Evren , E. Ringqvist , J. Doisne , A. Thaller , N. Sleiers , R. A. Flavell , J. P. Di Santo , T. Willinger , J. Exp. Med. 2022, 219, e20210987. 35019940 10.1084/jem.20210987 PMC8759608

[46] M. Zhang , M. Yang , T. Morimoto , N. Tajima , K. Ichiraku , K. Fujita , S. Iijima , M. Yudasaka , T. Okazaki , Carbon 2018, 127, 93.

[47] J. Y. Tse , A. Koike , K. Kadota , H. Uchiyama , K. Fujimori , Y. Tozuka , Eur. J. Pharm. Biopharm. 2021, 167, 116. 34363979 10.1016/j.ejpb.2021.07.017

[48] S. Carregal‐Romero , H. Groult , O. Canadas , N. A‐Gonzalez , A. V. Lechuga‐Vieco , B. Garcia‐Fojeda , F. Herranz , J. Pellico , A. Hidalgo , C. Casals , J. Ruiz‐Cabello , Biomater. Adv. 2022, 134, 112551. 35513950 10.1016/j.msec.2021.112551

[49] S. Attias Cohen , P. S. Kingma , J. A. Whitsett , R. Goldbart , T. Traitel , J. Kost , Int. J. Pharm. 2020, 585, 119387. 32473376 10.1016/j.ijpharm.2020.119387

[50] A. M. Shen , T. Minko , J. Control. Release. 2020, 326, 222. 32681948 10.1016/j.jconrel.2020.07.011 PMC7501141

[51] A. Wadhwa , A. Aljabbari , A. Lokras , C. Foged , A. Thakur , Pharmaceutics 2020, 12, 102. 32013049 10.3390/pharmaceutics12020102 PMC7076378

[52] Q. Chen , Y. Zhang , H. Yin , Adv. Drug Delivery Rev. 2021, 168, 246. 10.1016/j.addr.2020.10.014 33122087

[53] E. Frederix , A. K. Kuczaj , M. Nordlund , M. Bělka , F. Lizal , J. Jedelský , J. Elcner , M. Jícha , B. J. Geurts , J. Aerosol Sci. 2018, 115, 29.

[54] B. Asgharian , T. P. Owen , E. D. Kuempel , A. M. Jarabek , Toxicol. Appl. Pharmacol. 2018, 361, 27. 29738812 10.1016/j.taap.2018.05.001 PMC6329593

[55] M. Weiss , J. Fan , M. Claudel , T. Sonntag , P. Didier , C. Ronzani , L. Lebeau , F. Pons , J. Nanobiotechnology. 2021, 19, 5. 33407567 10.1186/s12951-020-00747-7 PMC7789233

[56] F. Mousseau , J. Berret , Soft Matter 2018, 14, 5764. 29989135 10.1039/c8sm00925b

[57] H. Majid , P. Madl , W. Hofmann , K. Alam , Aerosol Sci. Technol. 2012, 46, 547.

[58] H. Zhang , T. F. Bahamondez‐Canas , Y. Zhang , J. Leal , H. D. C. Smyth , Mol. Pharmaceutics 2018, 15, 4814. 10.1021/acs.molpharmaceut.8b00434 PMC6453125 30222933

[59] H. Zhang , Y. Zhang , R. O. 3. Williams , H. D. C. Smyth , Int. J. Pharm. 2021, 605, 120831. 34175380 10.1016/j.ijpharm.2021.120831

[60] S. Ni , Y. Liu , Y. Tang , J. Chen , S. Li , J. Pu , L. Han , Carbohydr. Polym. 2018, 179, 135. 29111036 10.1016/j.carbpol.2017.09.075

[61] J. Peng , Z. Cai , Q. Wang , J. Zhou , J. Xu , D. Pan , T. Chen , G. Zhang , L. Tao , Y. Chen , X. Shen , Molecules 2022, 27, 3610. 35684546 10.3390/molecules27113610 PMC9182538

[62] H. Li , J. Birchall , Pharm. Res. 2006, 23, 941. 16715384 10.1007/s11095-006-0027-x

[63] K. Fukushige , T. Tagami , M. Naito , E. Goto , S. Hirai , N. Hatayama , H. Yokota , T. Yasui , Y. Baba , T. Ozeki , Int. J. Pharm. 2020, 583, 119338. 32311468 10.1016/j.ijpharm.2020.119338

[64] K. Fukushige , T. Tagami , T. Ozeki , Journal of Drug Delivery Science and Technology 2017, 39, 435.

[65] T. Ito , M. Fukuhara , T. Okuda , H. Okamoto , Int. J. Pharm. 2020, 574, 118880. 31811928 10.1016/j.ijpharm.2019.118880

[66] R. Kandil , Y. Xie , R. Heermann , L. Isert , K. Jung , A. Mehta , O. M. Merkel , Adv. Ther. 2019, 2, 1900047. 10.1002/adtp.201900047 PMC6675603 31372493

[67] R. Kandil , D. Baldassi , S. Bohlen , J. T. Muller , D. C. Jurgens , T. Bargmann , S. Dehmel , Y. Xie , A. Mehta , K. Sewald , O. M. Merkel , J. Control. Release. 2023, 354, 305. 36634709 10.1016/j.jconrel.2023.01.014 PMC7614985

[68] Q. Ji , J. Hou , X. Yong , G. Gong , M. Muddassir , T. Tang , J. Xie , W. Fan , X. Chen , Adv. Mater. 2021, 33, 2007798. 10.1002/adma.202007798 33604928

[69] T. W. M. Keil , D. P. Feldmann , G. Costabile , Q. Zhong , S. da Rocha , O. M. Merkel , Eur. J. Pharm. Biopharm. 2019, 143, 61. 31445157 10.1016/j.ejpb.2019.08.012 PMC7611027

[70] M. Motiei , O. Mišík , T. H. Truong , F. Lizal , P. Humpolíček , V. Sedlařík , P. Sáha , Discover Nano 2023, 18, 38. 37382704 10.1186/s11671-023-03781-0 PMC10214903

[71] A. Bohr , N. Tsapis , C. Foged , I. Andreana , M. Yang , E. Fattal , Eur. J. Pharm. Biopharm. 2020, 156, 114. 32798665 10.1016/j.ejpb.2020.08.009 PMC7425770

[72] E. Bielski , Q. Zhong , H. Mirza , M. Brown , A. Molla , T. Carvajal , S. R. P. da Rocha , Int. J. Pharm. 2017, 527, 171. 28549971 10.1016/j.ijpharm.2017.05.046

[73] F. Joubert , M. J. Munson , A. Sabirsh , R. M. England , M. Hemmerling , C. Alexander , M. B. Ashford , J. Control. Release. 2023, 356, 580. 36918085 10.1016/j.jconrel.2023.03.011

[74] Y. Liu , Y. Li , D. Keskin , L. Shi , Adv. Healthcare Mater. 2019, 8, e1801359. 10.1002/adhm.201801359 30549448

[75] P. Mastorakos , A. L. da Silva , J. Chisholm , E. Song , W. K. Choi , M. P. Boyle , M. M. Morales , J. Hanes , J. S. Suk , Proc. Natl. Acad. Sci. USA 2015, 112, 8720. 26124127 10.1073/pnas.1502281112 PMC4507234

[76] L. Rotolo , D. Vanover , N. C. Bruno , H. E. Peck , C. Zurla , J. Murray , R. K. Noel , L. O'Farrell , M. Arainga , N. Orr‐Burks , J. Y. Joo , L. C. S. Chaves , Y. Jung , J. Beyersdorf , S. Gumber , R. Guerrero‐Ferreira , S. Cornejo , M. Thoresen , A. K. Olivier , K. M. Kuo , J. C. Gumbart , A. R. Woolums , F. Villinger , E. R. Lafontaine , R. J. Hogan , M. G. Finn , P. J. Santangelo , Nat. Mater. 2023, 22, 369. 36443576 10.1038/s41563-022-01404-0

[77] C. J. McKinlay , N. L. Benner , O. A. Haabeth , R. M. Waymouth , P. A. Wender , Proc. Natl. Acad. Sci. USA 2018, 115, E5859. 29891683 10.1073/pnas.1805358115 PMC6042134

[78] O. A. W. Haabeth , J. J. K. Lohmeyer , A. Sallets , T. R. Blake , I. Sagiv‐Barfi , D. K. Czerwinski , B. McCarthy , A. E. Powell , P. A. Wender , R. M. Waymouth , R. Levy , ACS Cent. Sci. 2021, 7, 1191. 34341771 10.1021/acscentsci.1c00361 PMC8265720

[79] N. L. Benner , R. L. McClellan , C. R. Turlington , O. A. W. Haabeth , R. M. Waymouth , P. A. Wender , J. Am. Chem. Soc. 2019, 141, 8416. 31083999 10.1021/jacs.9b03154 PMC7209379

[80] T. R. Blake , O. A. W. Haabeth , A. Sallets , R. L. McClellan , T. J. Del Castillo , J. G. Vilches‐Moure , W. C. Ho , P. A. Wender , R. Levy , R. M. Waymouth , Bioconjug. Chem. 2023, 34, 673. 10.1021/acs.bioconjchem.3c00019 PMC10601965 36996808

[81] G. M. Ryan , L. M. Kaminskas , B. D. Kelly , D. J. Owen , M. P. McIntosh , C. J. H. Porter , Mol. Pharmaceutics 2013, 10, 2986. 10.1021/mp400091n 23750747

[82] Y. Park , A. S. Moses , A. A. Demessie , P. Singh , H. Lee , T. Korzun , O. R. Taratula , A. W. G. Alani , O. Taratula , Mol. Pharmaceutics 2022, 19, 4696. 10.1021/acs.molpharmaceut.2c00738 PMC9826779 36409995

[83] G. Osman , J. Rodriguez , S. Y. Chan , J. Chisholm , G. Duncan , N. Kim , A. L. Tatler , K. M. Shakesheff , J. Hanes , J. S. Suk , J. E. Dixon , J. Control. Release. 2018, 285, 35. 30004000 10.1016/j.jconrel.2018.07.001 PMC6573017

[84] Y. Qiu , M. Y. T. Chow , W. Liang , W. W. Y. Chung , J. C. W. Mak , J. K. W. Lam , Mol. Pharmaceutics 2017, 14, 4606. 10.1021/acs.molpharmaceut.7b00725 29121767

[85] Y. Qiu , R. C. H. Man , Q. Liao , K. L. K. Kung , M. Y. T. Chow , J. K. W. Lam , J. Control. Release. 2019, 314, 102. 31629037 10.1016/j.jconrel.2019.10.026

[86] W. Liang , P. C. L. Kwok , M. Y. T. Chow , P. Tang , A. J. Mason , H. Chan , J. K. W. Lam , Eur. J. Pharm. Biopharm. 2014, 86, 64. 23702276 10.1016/j.ejpb.2013.05.006 PMC3948054

[87] W. Liang , M. Y. T. Chow , P. N. Lau , Q. T. Zhou , P. C. L. Kwok , G. P. H. Leung , A. J. Mason , H. Chan , L. L. M. Poon , J. K. W. Lam , Mol. Pharmaceutics 2015, 12, 910. 10.1021/mp500745v 25599953

[88] S. Fuchs , J. Klier , A. May , G. Winter , C. Coester , H. Gehlen , J. Microencapsul. 2012, 29, 615. 22432849 10.3109/02652048.2012.668962

[89] H. Abdelrady , R. M. Hathout , R. Osman , I. Saleem , N. D. Mortada , Eur. J. Pharm. Sci. 2019, 133, 115. 30905615 10.1016/j.ejps.2019.03.016

[90] A. Mehta , E. Dalle Vedove , L. Isert , O. M. Merkel , Pharm. Res. 2019, 36, 133. 31289919 10.1007/s11095-019-2665-9 PMC7611029

[91] M. Y. T. Chow , Y. Qiu , Q. Liao , P. C. L. Kwok , S. F. Chow , H. Chan , J. K. W. Lam , Int. J. Pharm. 2019, 572, 118818. 31678379 10.1016/j.ijpharm.2019.118818

[92] R. Dalirfardouei , M. Tafaghodi , Z. Meshkat , A. Najafi , A. Gholoobi , M. S. Nabavinia , S. Sajedifar , M. Meshkat , A. Badiee , M. Ramezani , A. Varasteh , M. Naderinasab , Iran. J. Basic Med. Sci. 2020, 23, 826. 32695300 10.22038/ijbms.2020.41806.9881 PMC7351443

[93] L. Gomes Dos Reis , W. Lee , M. Svolos , L. M. Moir , R. Jaber , A. Engel , N. Windhab , P. M. Young , D. Traini , Drug Dev. Ind. Pharm. 2020, 46, 427. 32070151 10.1080/03639045.2020.1724134

[94] T. Kurosaki , H. Kanda , J. Hashizume , K. Sato , H. Harasawa , T. Nakamura , H. Sasaki , Y. Kodama , Pharmaceutics 2021, 13, 1983. 34834398 10.3390/pharmaceutics13111983 PMC8625672

[95] Y. Hagino , I. A. Khalil , S. Kimura , K. Kusumoto , H. Harashima , Mol. Pharmaceutics 2021, 18, 878. 10.1021/acs.molpharmaceut.0c00854 33492961

[96] S. M. Johler , J. Rejman , S. Guan , J. Rosenecker , PLoS One 2015, 10, e0137504. 26352268 10.1371/journal.pone.0137504 PMC4564175

[97] K. Thanki , D. van Eetvelde , A. Geyer , J. Fraire , R. Hendrix , H. Van Eygen , E. Putteman , H. Sami , C. de Souza Carvalho‐Wodarz , H. Franzyk , H. M. Nielsen , K. Braeckmans , C. Lehr , M. Ogris , C. Foged , J. Control. Release. 2019, 310, 82. 31398360 10.1016/j.jconrel.2019.08.004

[98] J. Wang , M. S. Hanafy , H. Xu , J. Leal , Y. Zhai , D. Ghosh , R. O. Williams Iii , H. D C Smyth , Z. Cui , Int. J. Pharm. 2021, 596, 120215. 33486021 10.1016/j.ijpharm.2021.120215

[99] Y. Hattori , K. Tamaki , K. Ozaki , K. Kawano , H. Onishi , Journal of Drug Delivery Science and Technology 2019, 52, 1042.

[100] S. Subramaniam , P. Joyce , L. Donnellan , C. Young , A. Wignall , P. Hoffmann , C. A. Prestidge , J. Colloid Interface Sci. 2023, 641, 36. 36924544 10.1016/j.jcis.2023.03.048

[101] R. Kanasty , J. R. Dorkin , A. Vegas , D. Anderson , Nat. Mater. 2013, 12, 967. 24150415 10.1038/nmat3765

[102] J. E. Dahlman , C. Barnes , O. Khan , A. Thiriot , S. Jhunjunwala , T. E. Shaw , Y. Xing , H. B. Sager , G. Sahay , L. Speciner , A. Bader , R. L. Bogorad , H. Yin , T. Racie , Y. Dong , S. Jiang , D. Seedorf , A. Dave , K. S. Sandu , M. J. Webber , T. Novobrantseva , V. M. Ruda , A. K. R. Lytton‐Jean , C. G. Levins , B. Kalish , D. K. Mudge , M. Perez , L. Abezgauz , P. Dutta , L. Smith , et al., Nat. Nanotechnol. 2014, 9, 648. 24813696 10.1038/nnano.2014.84 PMC4207430

[103] M. P. Lokugamage , D. Vanover , J. Beyersdorf , M. Z. C. Hatit , L. Rotolo , E. S. Echeverri , H. E. Peck , H. Ni , J. K. Yoon , Y. Kim , P. J. Santangelo , J. E. Dahlman , Nat. Biomed. Eng. 2021, 5, 1059. 34616046 10.1038/s41551-021-00786-x PMC10197923

[104] B. Li , R. S. Manan , S. Liang , A. Gordon , A. Jiang , A. Varley , G. Gao , R. Langer , W. Xue , D. Anderson , Nat. Biotechnol. 2023, 41, 1410. 36997680 10.1038/s41587-023-01679-x PMC10544676

[105] J. Holtzman , H. Lee , Exp. Mol. Med. 2020, 52, 887. 32541816 10.1038/s12276-020-0450-9 PMC7338515

[106] Y. Han , Y. Zhu , H. A. Youngblood , S. Almuntashiri , T. W. Jones , X. Wang , Y. Liu , P. R. Somanath , D. Zhang , J. Control. Release. 2022, 352, 556. 36341934 10.1016/j.jconrel.2022.10.052 PMC13092262

[107] P. C. Dinh , D. Paudel , H. Brochu , K. D. Popowski , M. C. Gracieux , J. Cores , K. Huang , M. T. Hensley , E. Harrell , A. C. Vandergriff , A. K. George , R. T. Barrio , S. Hu , T. A. Allen , K. Blackburn , T. G. Caranasos , X. Peng , L. V. Schnabel , K. B. Adler , L. J. Lobo , M. B. Goshe , K. Cheng , Nat. Commun. 2020, 11, 1064. 32111836 10.1038/s41467-020-14344-7 PMC7048814

[108] Z. Wang , K. D. Popowski , D. Zhu , B. L. de Juan Abad , X. Wang , M. Liu , H. Lutz , N. De Naeyer , C. T. DeMarco , T. N. Denny , P. C. Dinh , Z. Li , K. Cheng , Nat. Biomed. Eng. 2022, 6, 791. 35788687 10.1038/s41551-022-00902-5 PMC10782831

[109] K. D. Popowski , A. Moatti , G. Scull , D. Silkstone , H. Lutz , B. Lopez de Juan Abad , A. George , E. Belcher , D. Zhu , X. Mei , X. Cheng , M. Cislo , A. Ghodsi , Y. Cai , K. Huang , J. Li , A. C. Brown , A. Greenbaum , P. C. Dinh , K. Cheng , Matter 2022, 5, 2960. 35847197 10.1016/j.matt.2022.06.012 PMC9272513

[110] G. A. Duncan , J. Jung , J. Hanes , J. S. Suk , Mol. Ther. 2016, 24, 2043. 27646604 10.1038/mt.2016.182 PMC5167788

[111] G. A. Duncan , N. Kim , Y. Colon‐Cortes , J. Rodriguez , M. Mazur , S. E. Birket , S. M. Rowe , N. E. West , A. Livraghi‐Butrico , R. C. Boucher , J. Hanes , G. Aslanidi , J. S. Suk , Mol. Ther. Methods Clin. Dev. 2018, 9, 296. 30038933 10.1016/j.omtm.2018.03.006 PMC6054694

[112] S. Qin , H. Wang , G. Liu , H. Mei , M. Chen , Mol. Med. Rep. 2019, 20, 4953. 31702805 10.3892/mmr.2019.10779 PMC6854583

[113] C. M. Zimmermann , D. Baldassi , K. Chan , N. B. P. Adams , A. Neumann , D. L. Porras‐Gonzalez , X. Wei , N. Kneidinger , M. G. Stoleriu , G. Burgstaller , D. Witzigmann , P. Luciani , O. M. Merkel , J. Control. Release. 2022, 351, 137. 36126785 10.1016/j.jconrel.2022.09.021 PMC7613708

[114] T. W. Keil , C. Zimmermann , D. Baldassi , F. Adams , W. Friess , A. Mehta , O. M. Merkel , Adv. Ther. 2021, 4, 2100073. 10.1002/adtp.202100073 PMC7611418 34337144

[115] E. Daviskas , B. K. Rubin , Expert Rev. Respir. Med. 2013, 7, 65. 23362816 10.1586/ers.12.72

[116] A. Kumari , S. Pal , B. R. G. F. P. Mohny , N. Gupta , C. Miglani , B. Pattnaik , A. Pal , M. Ganguli , Mol. Pharmaceutics 2022, 19, 1309. 10.1021/acs.molpharmaceut.1c00770 35333535

[117] M. Agnoletti , A. Bohr , K. Thanki , F. Wan , X. Zeng , J. P. Boetker , M. Yang , C. Foged , Eur. J. Pharm. Biopharm. 2017, 120, 9. 28780275 10.1016/j.ejpb.2017.08.001

[118] H. Miao , K. Huang , Y. Li , R. Li , X. Zhou , J. Shi , Z. Tong , Z. Sun , A. Yu , Int. J. Pharm. 2023, 640, 123050. 37201764 10.1016/j.ijpharm.2023.123050

[119] Y. Xu , L. Harinck , A. G. Lokras , P. Gerde , E. Selg , C. Sjoberg , H. Franzyk , A. Thakur , C. Foged , Int. J. Pharm. 2022, 621, 121758. 35483619 10.1016/j.ijpharm.2022.121758

[120] A. Mohamed , A. Y. Pekoz , K. Ross , G. A. Hutcheon , I. Y. Saleem , Int. J. Pharm. 2019, 569, 118524. 31319144 10.1016/j.ijpharm.2019.118524

[121] H. Li , P. C. Seville , I. J. Williamson , J. C. Birchall , J. Gene Med. 2005, 7, 343. 15515142 10.1002/jgm.654

[122] E. Potocka , J. P. Cassidy , P. Haworth , D. Heuman , S. van Marle , R. A. J. Baughman , J. Diabetes Sci. Technol. 2010, 4, 1164. 20920436 10.1177/193229681000400515 PMC2956823

[123] Q. Wang , G. Mi , D. Hickey , Y. Li , J. Tu , T. J. Webster , Y. Shen , Biomaterials 2018, 160, 107. 29407340 10.1016/j.biomaterials.2018.01.022

[124] Q. Wang , Y. Shen , G. Mi , D. He , Y. Zhang , Y. Xiong , T. J. Webster , J. Tu , Biomaterials 2020, 228, 119575. 31677394 10.1016/j.biomaterials.2019.119575

[125] A. Fernandez Tena , P. Casan Clara , Arch. Bronconeumol. 2012, 48, 240. 22464044 10.1016/j.arbres.2012.02.003

[126] J. Wu , L. Wu , F. Wan , J. Rantanen , D. Cun , M. Yang , Int. J. Pharm. 2019, 566, 32. 31077763 10.1016/j.ijpharm.2019.05.019

[127] M. Y. T. Chow , Y. Qiu , F. F. K. Lo , H. H. S. Lin , H. Chan , P. C. L. Kwok , J. K. W. Lam , Int. J. Pharm. 2017, 530, 40. 28720537 10.1016/j.ijpharm.2017.07.013

[128] C. Dormenval , A. Lokras , G. Cano‐Garcia , A. Wadhwa , K. Thanki , F. Rose , A. Thakur , H. Franzyk , C. Foged , Pharm. Res. 2019, 36, 142. 31376020 10.1007/s11095-019-2663-y

[129] D. K. Jensen , L. B. Jensen , S. Koocheki , L. Bengtson , D. Cun , H. M. Nielsen , C. Foged , J. Control. Release. 2012, 157, 141. 21864597 10.1016/j.jconrel.2011.08.011

[130] D. P. Gaspar , J. Vital , M. C. Leiva , L. M. Goncalves , P. Taboada , C. Remunan‐Lopez , J. Vitor , A. J. Almeida , Nanomedicine 2019, 14, 407. 30698066 10.2217/nnm-2018-0270

[131] W. Liang , M. Y. T. Chow , S. F. Chow , H. Chan , P. C. L. Kwok , J. K. W. Lam , Int. J. Pharm. 2018, 552, 67. 30244146 10.1016/j.ijpharm.2018.09.045

[132] W. Liang , A. Y. L. Chan , M. Y. T. Chow , F. F. K. Lo , Y. Qiu , P. C. L. Kwok , J. K. W. Lam , Asian J. Pharm. Sci. 2018, 13, 163. 32104389 10.1016/j.ajps.2017.10.002 PMC7032260

[133] Y. Yan , Q. Wu , P. Ren , Q. Liu , N. Zhang , Y. Ji , J. Liu , Int. J. Biol. Macromol. 2021, 193, 1043. 34800517 10.1016/j.ijbiomac.2021.11.088

[134] Y. Li , M. Han , T. Liu , D. Cun , L. Fang , M. Yang , Carbohydr. Polym. 2017, 172, 197. 28606525 10.1016/j.carbpol.2017.05.020

[135] D. Shen , Y. Shen , Q. Chen , B. Huang , Y. Mi , Y. Shan , G. Yu , T. J. Webster , Nanomedicine 2020, 15, 489. 32077793 10.2217/nnm-2019-0376

[136] H. Lv , T. Wang , F. Ma , K. Zhang , T. Gao , R. Pei , Y. Zhang , Biomed. Mater. 2022, 17, 024108. 10.1088/1748-605X/ac5415 35147520

[137] B. S. Schuster , J. S. Suk , G. F. Woodworth , J. Hanes , Biomaterials 2013, 34, 3439. 23384790 10.1016/j.biomaterials.2013.01.064 PMC3590854

[138] P. Ravikumar , J. U. Menon , P. Punnakitikashem , D. Gyawali , O. Togao , M. Takahashi , J. Zhang , J. Ye , O. W. Moe , K. T. Nguyen , C. C. W. Hsia , Nanomedicine 2016, 12, 811. 26518603 10.1016/j.nano.2015.10.004 PMC4809756

[139] M. K. Grun , A. Suberi , K. Shin , T. Lee , V. Gomerdinger , Z. M. Moscato , A. S. Piotrowski‐Daspit , W. M. Saltzman , Biomaterials 2021, 272, 120780. 33813260 10.1016/j.biomaterials.2021.120780 PMC8085770

[140] N. Kim , G. Kwak , J. Rodriguez , A. Livraghi‐Butrico , X. Zuo , V. Simon , E. Han , S. K. Shenoy , N. Pandey , M. Mazur , S. E. Birket , A. Kim , S. M. Rowe , R. Boucher , J. Hanes , J. S. Suk , Thorax 2022, 77, 812. 34697091 10.1136/thoraxjnl-2020-215185 PMC9129924

[141] S. Currie , S. Kim , X. Gu , X. Ren , F. Lin , S. Liu , C. Yang , J. Kim , S. Liu , Colloids Surf. B Biointerfaces. 2020, 196, 111287. 32768985 10.1016/j.colsurfb.2020.111287

[142] G. Conte , G. Costabile , D. Baldassi , V. Rondelli , R. Bassi , D. Colombo , G. Linardos , E. V. Fiscarelli , R. Sorrentino , A. Miro , F. Quaglia , P. Brocca , I. d'Angelo , O. M. Merkel , F. Ungaro , ACS Appl. Mater. Interfaces. 2022, 14, 7565. 35107987 10.1021/acsami.1c14975 PMC8855343

[143] S. Guan , A. Munder , S. Hedtfeld , P. Braubach , S. Glage , L. Zhang , S. Lienenklaus , A. Schultze , G. Hasenpusch , W. Garrels , F. Stanke , C. Miskey , S. M. Johler , Y. Kumar , B. Tummler , C. Rudolph , Z. Ivics , J. Rosenecker , Nat. Nanotechnol. 2019, 14, 287. 30692673 10.1038/s41565-018-0358-x

[144] S. Sun , E. Li , G. Zhao , J. Tang , Q. Zuo , L. Cai , C. Xu , C. Sui , Y. Ou , C. Liu , H. Li , Y. Ding , C. Li , D. Lu , W. Zhang , P. Luo , P. Cheng , Y. Gao , C. Tu , B. Pitard , J. Rosenecker , B. Wang , Y. Liu , Q. Zou , S. Guan , Biomaterials 2023, 292, 121907. 36436305 10.1016/j.biomaterials.2022.121907 PMC9673044

[145] J. Leal , X. Peng , X. Liu , D. Arasappan , D. C. Wylie , S. H. Schwartz , J. J. Fullmer , B. C. McWilliams , H. D. C. Smyth , D. Ghosh , J. Control. Release. 2020, 322, 457. 32243979 10.1016/j.jconrel.2020.03.032 PMC7250722

[146] H. Wang , Y. Wang , Y. Wang , J. Hu , T. Li , H. Liu , Q. Zhang , Y. Cheng , Angew. Chem. Int. Ed Engl. 2015, 54, 11647. 26260847 10.1002/anie.201501461

[147] L. Wang , D. Wu , H. Xu , Y. You , Angew. Chem. Int. Ed Engl. 2016, 55, 755. 26586102 10.1002/anie.201508695

[148] C. Ge , J. Yang , S. Duan , Y. Liu , F. Meng , L. Yin , Nano Lett. 2020, 20, 1738. 32039603 10.1021/acs.nanolett.9b04957

[149] R. Charbaji , M. Kar , L. E. Theune , J. Bergueiro , A. Eichhorst , L. Navarro , P. Graff , F. Stumpff , M. Calderon , S. Hedtrich , Small 2021, 17, e2007963. 33719187 10.1002/smll.202007963

[150] A. Sharma , K. Vaghasiya , P. Gupta , A. K. Singh , U. D. Gupta , R. K. Verma , J. Control. Release. 2020, 324, 17. 32418903 10.1016/j.jconrel.2020.05.013

[151] D. P. Ferreira , S. V. Martini , H. A. Oliveira , A. L. Silva , S. Shenoy , D. Chen , V. Simon , E. Han , N. E. West , J. S. Suk , P. R. M. Rocco , H. Petrs‐Silva , M. M. Morales , F. F. Cruz , Cell. Physiol. Biochem. 2023, 57, 331. 37724045 10.33594/000000660 PMC13156840

[152] C. Bao , B. Liu , B. Li , J. Chai , L. Zhang , L. Jiao , D. Li , Z. Yu , F. Ren , X. Shi , Y. Li , Nano Lett. 2020, 20, 1352. 31904988 10.1021/acs.nanolett.9b04841

[153] L. M. P. Vermeulen , S. C. De Smedt , K. Remaut , K. Braeckmans , Eur. J. Pharm. Biopharm. 2018, 129, 184. 29859281 10.1016/j.ejpb.2018.05.034

[154] Z. Ma , S. W. Wong , H. Forgham , L. Esser , M. Lai , M. N. Leiske , K. Kempe , G. Sharbeen , J. Youkhana , F. Mansfeld , J. F. Quinn , P. A. Phillips , T. P. Davis , M. Kavallaris , J. A. McCarroll , Biomaterials 2022, 285, 121539. 35500393 10.1016/j.biomaterials.2022.121539

[155] T. Miyazaki , S. Uchida , S. Nagatoishi , K. Koji , T. Hong , S. Fukushima , K. Tsumoto , K. Ishihara , K. Kataoka , H. Cabral , Adv. Healthcare Mater. 2020, 9, e2000538. 10.1002/adhm.202000538 32583633

[156] N. Gong , Y. Zhang , X. Teng , Y. Wang , S. Huo , G. Qing , Q. Ni , X. Li , J. Wang , X. Ye , T. Zhang , S. Chen , Y. Wang , J. Yu , P. C. Wang , Y. Gan , J. Zhang , M. J. Mitchell , J. Li , X. Liang , Nat. Nanotechnol. 2020, 15, 1053. 33106640 10.1038/s41565-020-00782-3 PMC7719078

[157] H. Wang , L. Qin , X. Zhang , J. Guan , S. Mao , J. Control. Release. 2022, 352, 970. 36372386 10.1016/j.jconrel.2022.10.061 PMC9671523

[158] S. Liu , Q. Cheng , T. Wei , X. Yu , L. T. Johnson , L. Farbiak , D. J. Siegwart , Nat. Mater. 2021, 20, 701. 33542471 10.1038/s41563-020-00886-0 PMC8188687

[159] A. Mukherjee , K. D. MacDonald , J. Kim , M. I. Henderson , Y. Eygeris , G. Sahay , Sci. Adv. 2020, 6, eabc5911. 33208364 10.1126/sciadv.abc5911 PMC7673816

[160] H. Jin , M. Jeong , G. Lee , M. Kim , Y. Yoo , H. J. Kim , J. Cho , Y. Lee , H. Lee , Adv. Funct. Mater. 2023, 33, 2209432.

[161] A. M. Hafner , B. Corthesy , H. P. Merkle , Adv. Drug Delivery Rev. 2013, 65, 1386. 10.1016/j.addr.2013.05.013 23751781

[162] S. Rauch , J. Lutz , A. Kowalczyk , T. Schlake , R. Heidenreich , Methods Mol. Biol. 2017, 1499, 89. 27987144 10.1007/978-1-4939-6481-9_5

[163] J. Xu , H. Wang , L. Xu , Y. Chao , C. Wang , X. Han , Z. Dong , H. Chang , R. Peng , Y. Cheng , Z. Liu , Biomaterials 2019, 207, 1. 30947117 10.1016/j.biomaterials.2019.03.037

[164] T. Frohlich , D. Edinger , R. Klager , C. Troiber , E. Salcher , N. Badgujar , I. Martin , D. Schaffert , A. Cengizeroglu , P. Hadwiger , H. Vornlocher , E. Wagner , J. Control. Release. 2012, 160, 532. 22465674 10.1016/j.jconrel.2012.03.018

[165] S. Uchida , H. Kinoh , T. Ishii , A. Matsui , T. A. Tockary , K. M. Takeda , H. Uchida , K. Osada , K. Itaka , K. Kataoka , Biomaterials 2016, 82, 221. 26763736 10.1016/j.biomaterials.2015.12.031

[166] A. Krhac Levacic , S. Berger , J. Muller , A. Wegner , U. Lachelt , C. Dohmen , C. Rudolph , E. Wagner , J. Control. Release. 2021, 339, 27. 34547258 10.1016/j.jconrel.2021.09.016

[167] H. J. Kim , S. Ogura , T. Otabe , R. Kamegawa , M. Sato , K. Kataoka , K. Miyata , ACS Cent. Sci. 2019, 5, 1866. 31807688 10.1021/acscentsci.9b00843 PMC6891845

[168] J. C. Kaczmarek , K. J. Kauffman , O. S. Fenton , K. Sadtler , A. K. Patel , M. W. Heartlein , F. DeRosa , D. G. Anderson , Nano Lett. 2018, 18, 6449. 30211557 10.1021/acs.nanolett.8b02917 PMC6415675

[169] T. Miyazaki , S. Uchida , H. Hatano , Y. Miyahara , A. Matsumoto , H. Cabral , Eur. Polym. J. 2020, 140, 110028.

[170] W. Yang , T. Miyazaki , Y. Nakagawa , E. Boonstra , K. Masuda , Y. Nakashima , P. Chen , L. Mixich , K. Barthelmes , A. Matsumoto , P. Mi , S. Uchida , H. Cabral , Sci. Technol. Adv. Mater. 2023, 24, 2170164. 36950277 10.1080/14686996.2023.2170164 PMC10026751

[171] N. Yoshinaga , S. Uchida , M. Naito , K. Osada , H. Cabral , K. Kataoka , Biomaterials 2019, 197, 255. 30669016 10.1016/j.biomaterials.2019.01.023

[172] N. Yoshinaga , S. Uchida , A. Dirisala , M. Naito , K. Koji , K. Osada , H. Cabral , K. Kataoka , Adv. Healthcare Mater. 2022, 11, e2102016. 10.1002/adhm.202102016 34913604

[173] S. Vaidyanathan , K. T. Azizian , A. K. M. A. Haque , J. M. Henderson , A. Hendel , S. Shore , J. S. Antony , R. I. Hogrefe , M. S. D. Kormann , M. H. Porteus , A. P. McCaffrey , Mol. Ther. Nucleic Acids. 2018, 12, 530. 30195789 10.1016/j.omtn.2018.06.010 PMC6076213

[174] K. Kariko , H. Muramatsu , F. A. Welsh , J. Ludwig , H. Kato , S. Akira , D. Weissman , Mol. Ther. 2008, 16, 1833. 18797453 10.1038/mt.2008.200 PMC2775451

[175] B. Li , X. Luo , Y. Dong , Bioconjug. Chem. 2016, 27, 849. 26906521 10.1021/acs.bioconjchem.6b00090

[176] J. Nelson , E. W. Sorensen , S. Mintri , A. E. Rabideau , W. Zheng , G. Besin , N. Khatwani , S. V. Su , E. J. Miracco , W. J. Issa , S. Hoge , M. G. Stanton , J. L. Joyal , Sci. Adv. 2020, 6, eaaz6893. 32637598 10.1126/sciadv.aaz6893 PMC7314518

[177] D. M. Mauger , B. J. Cabral , V. Presnyak , S. V. Su , D. W. Reid , B. Goodman , K. Link , N. Khatwani , J. Reynders , M. J. Moore , I. J. McFadyen , Proc. Natl. Acad. Sci. USA 2019, 116, 24075. 31712433 10.1073/pnas.1908052116 PMC6883848

[178] Y. Oe , R. J. Christie , M. Naito , S. A. Low , S. Fukushima , K. Toh , Y. Miura , Y. Matsumoto , N. Nishiyama , K. Miyata , K. Kataoka , Biomaterials 2014, 35, 7887. 24930854 10.1016/j.biomaterials.2014.05.041

[179] H. Takemoto , K. Miyata , S. Hattori , T. Ishii , T. Suma , S. Uchida , N. Nishiyama , K. Kataoka , Angew. Chem. Int. Ed Engl. 2013, 52, 6218. 23630117 10.1002/anie.201300178

[180] H. Park , E. Bang , J. J. Hong , S. Lee , H. L. Ko , H. W. Kwak , H. Park , K. W. Kang , R. Kim , S. R. Ryu , G. Kim , H. Oh , H. Kim , K. Lee , M. Kim , S. Y. Kim , J. Kim , K. El‐Baz , H. Lee , M. Song , D. G. Jeong , G. Keum , J. Nam , Angew. Chem. Int. Ed Engl. 2020, 59, 11540. 32239636 10.1002/anie.202002979

[181] A. Dirisala , S. Uchida , T. A. Tockary , N. Yoshinaga , J. Li , S. Osawa , L. Gorantla , S. Fukushima , K. Osada , K. Kataoka , J. Drug Target. 2019, 27, 670. 30499743 10.1080/1061186X.2018.1550646

[182] S. Uchida , Y. Yamaberi , M. Tanaka , M. Oba , Nanoscale 2021, 13, 18941. 34664600 10.1039/d1nr03600a

[183] D. S. Manickam , J. Li , D. A. Putt , Q. Zhou , C. Wu , L. H. Lash , D. Oupicky , J. Control. Release. 2010, 141, 77. 19720098 10.1016/j.jconrel.2009.08.022 PMC2791204

[184] M. Naito , Y. Otsu , R. Kamegawa , K. Hayashi , S. Uchida , H. J. Kim , K. Miyata , Sci. Technol. Adv. Mater. 2019, 20, 105. 30787961 10.1080/14686996.2019.1569818 PMC6374946

[185] L. Schoenmaker , D. Witzigmann , J. A. Kulkarni , R. Verbeke , G. Kersten , W. Jiskoot , D. J. A. Crommelin , Int. J. Pharm. 2021, 601, 120586. 33839230 10.1016/j.ijpharm.2021.120586 PMC8032477

[186] P. Kolhe , E. Amend , S. K. Singh , Biotechnol. Prog. 2010, 26, 727. 20039442 10.1002/btpr.377

[187] K. L. Jones , D. Drane , E. J. Gowans , BioTechniques 2007, 43, 675. 18072597 10.2144/000112593 PMC4526277

[188] D. Shirane , H. Tanaka , Y. Nakai , H. Yoshioka , H. Akita , Biol. Pharm. Bull. 2018, 41, 1291. 30068880 10.1248/bpb.b18-00208

[189] D. Shirane , H. Tanaka , Y. Sakurai , S. Taneichi , Y. Nakai , K. Tange , I. Ishii , H. Akita , Pharmaceutics 2023, 15, 1819. 37514007 10.3390/pharmaceutics15071819 PMC10383539

[190] H. Muramatsu , K. Lam , C. Bajusz , D. Laczko , K. Kariko , P. Schreiner , A. Martin , P. Lutwyche , J. Heyes , N. Pardi , Mol. Ther. 2022, 30, 1941. 35131437 10.1016/j.ymthe.2022.02.001 PMC8815268

[191] B. Kim , R. R. Hosn , T. Remba , D. Yun , N. Li , W. Abraham , M. B. Melo , M. Cortes , B. Li , Y. Zhang , Y. Dong , D. J. Irvine , J. Control. Release. 2023, 353, 241. 36414195 10.1016/j.jconrel.2022.11.022 PMC9708520

